# Multivalent Lactose–Ferrocene Conjugates Based on Poly (Amido Amine) Dendrimers and Gold Nanoparticles as Electrochemical Probes for Sensing Galectin-3

**DOI:** 10.3390/nano10020203

**Published:** 2020-01-24

**Authors:** Manuel C. Martos-Maldonado, Indalecio Quesada-Soriano, Luis García-Fuentes, Antonio Vargas-Berenguel

**Affiliations:** Department of Chemistry and Physics, University of Almería, Carretera de Sacramento s/n, 04120 Almería, Spain; mmm588@inlumine.ual.es (M.C.M.-M.); indaqs@ual.es (I.Q.-S.); lgarcia@ual.es (L.G.-F.)

**Keywords:** galectin-3, electrochemical probes, gold nanoparticles, ferrocene, electroactive glycodendrimers, PAMAM

## Abstract

Galectin-3 is considered a cancer biomarker and bioindicator of fibrosis and cardiac remodeling and, therefore, it is desirable to develop convenient methods for its detection. Herein, an approach based on the development of multivalent electrochemical probes with high galectin-3 sensing abilities is reported. The probes consist of multivalent presentations of lactose–ferrocene conjugates scaffolded on poly (amido amine) (PAMAM) dendrimers and gold nanoparticles. Such multivalent lactose–ferrocene conjugates are synthesized by coupling of azidomethyl ferrocene–lactose building blocks on alkyne-functionalized PAMAM, for the case of the glycodendrimers, and to disulfide-functionalized linkers that are then used for the surface modification of citrate-stabilized gold nanoparticles. The binding and sensing abilities toward galectin-3 of both ferrocene-containing lactose dendrimers and gold nanoparticles have been evaluated by means of isothermal titration calorimetry, UV–vis spectroscopy, and differential pulse voltammetry. The highest sensitivity by electrochemical methods to galectin-3 was shown by lactosylferrocenylated gold nanoparticles, which are able to detect the lectin in nanomolar concentrations.

## 1. Introduction

Galectins are a family of β-D-galactoside-binding lectins, which are widespread throughout the animal kingdom and are ubiquitous in adult humans. Among these lectins, galectin-3 (Gal-3) occupies a prominent place due to its implications in various physiological and pathological processes, such as inflammation, cancer progression, and metastasis through various mechanisms, including cell–cell adhesion, cell–matrix interactions, apoptosis, angiogenesis, and mRNA splicing. Gal-3 plays a key role in triggering inflammation in many acute and chronic conditions, including atherosclerosis and heart failure, autoimmune diseases, neurological degeneration, and diabetes, among others. Gal-3 is overexpressed in many tumor cells and is considered to be a cancer biomarker [1]. Furthermore, high blood Gal-3 levels have been correlated with the likelihood of heart failure, and the protein is a novel bioindicator of fibrosis and cardiac remodeling [2,3,4,5,6,7]. Therefore, there is a need for the development of Gal-3 detection and monitoring methods in serum to provide useful information on cancer progression and cardiovascular risk. Such methods should meet the characteristics of convenience, inexpensiveness, and robustness.

Most of the reported assay methods are based on routine immunoassays [8,9], Western blot analysis or an enzyme-linked immunosorbent assay (ELISA) [10,11,12], and immunohistochemical methods [13,14]. However, despite the advantages of immunoassay-type methods, they exhibit many inherent limitations; they can be labor-intensive, expensive, and suffer from a lack of reproducibility, a high possibility of false positive/negative results, and a lack of stability of antibodies and enzymes [15,16].

To improve Gal-3 detection sensitivity and reproducibility, immunoassay-type methods can be combined with other techniques. For example, a sandwich-type electrochemical immunosensor that uses Gal-3 antibodies has been reported recently [17]. This method is based on MIL-88(Fe) metal–organic framework/Au hybrid nanoparticles immobilized on *N*-doped graphene nanoribbons for the immobilization of the primary Gal-3 antibody and involves AuPt-methylene blue. The latter is a redox nanoprobe for the generation and amplification of electrochemical signals. Although direct detection of Gal-3 by means of surface plasmon resonance (SPR) measurements using lactose-modified gold surfaces has been reported [18], an optical biosensor that uses the anti-Gal-3 antibody as a biorecognition element has been developed for the quantification of Gal-3 based on a graphene oxide-enhanced SPR methodology for transducing the immunological interaction [19].

Efforts to develop alternative sensing methods include the use of galactose-containing chemical probes. A biotinylated *N-*acetyl lactosamine–polyacrylamide conjugate has been used as a probe for Gal-3 sensing in a sandwich-type assay. The immobilized lectin bound to the conjugate is detected by means of the enhanced chemiluminescence of horseradish peroxidase–streptavidin bound to the probe [20].

Synthesized molecular probes with an enhanced affinity to Gal-3 have been applied in optical Gal-3 sensing. This method consists of using ligands, such as lactose or thiodigalactoside derivatives bearing a photo label for covalent attachment to Gal-3 upon binding and photoirradiation, and an azido or propargyl group for the subsequent “clicking” of fluorescent derivatives for in-gel visualization of the protein [21,22,23,24].

Furthermore, the synthesis of a lactose derivative containing a lanthanide-binding tag allows for the use of such ligands as a molecular probe for sensing Gal-3 through NMR measurements, thereby exploiting the paramagnetic effects of the presence of the lanthanide cation [25].

However, the development of electrochemical probes for the detection of Gal-3 is very scarce. So far, to the best of our knowledge, only two reported examples involve the use of electrochemical methods [17,26]. Of them, only one is based on the use of a Gal-3 ligand probe; that is, galactose-containing single-walled carbon nanotubes [26].

Electrochemical sensors have found a wide range of applications in industrial, environmental, clinical, agricultural, and food analysis. Such devices present some advantages. For example, they can be used for direct measurements in complex matrices, they are susceptible to miniaturization, and they can provide rapid, highly sensitive, reliable, and inexpensive measurements [27,28,29,30].

We previously reported on the redox and sensing ability properties of carbohydrate and peptide–ferrocene conjugates toward proteins [31,32,33,34,35,36,37,38,39]. An interesting finding was the fact that the multivalent presentation of ferrocene–mannose conjugates led to a remarkable enhancement of the protein sensing properties due to the multivalent glycoside effect and the multielectron exchange as a result of the multiple ferrocene units [36,38]. In addition, the electroactive mannosylated gold nanoparticles complemented their redox sensing abilities with the optical sensing abilities that derive from their plasmonic properties. In this paper, we use our approach to develop multivalent electrochemical probes with high Gal-3 sensing abilities. Since many lectins are oligomeric, a very successful strategy to obtain high-affinity ligands is the design of multivalent compounds. Nevertheless, Gal-3 is mainly present in solution in a monomeric form in rapid equilibrium with a small percentage of protein oligomer [40]. The Gal-3 structure comprises, in addition to the carbohydrate recognition domain (CRD), an *N*-terminal, and collagen-like domains that have demonstrated to be important for the formation of Gal-3 oligomers, though CRD is also claimed to be involved in inducing aggregation [4,40,41,42]. For example, the specific interaction of Gal-3 with multivalent carbohydrates induces the formation of cross-linked complexes involving Gal-3 pentamers [40]. In addition, dynamic light scattering (DLS) experiments have shown that lactose-functionalized poly (amido amine) (PAMAM) dendrimers of generation 2 to 6 induce Gal-3 aggregation [43]. Moreover, a multivalent effect was reported to arise from the enhanced binding affinity of glycoclusters bearing thiodigalactoside derivatives toward Gal-3 [44].

Our studies started with the synthesis of electroactive poly (amidoamine) (PAMAM)-based dendrimers from generation 0 to 2, as well as gold nanoparticles bearing lactosylferrocenyl moieties in the periphery. The binding and sensing abilities of such multivalent systems toward Gal-3 have been evaluated by means of isothermal titration calorimetry, UV–vis spectroscopy, and differential pulse voltammetry.

## 2. Materials and Methods

### 2.1. General Methods

Thin-layer chromatography (TLC) was performed on Merck silica gel 60 F254 aluminum sheets and developed by UV–vis light and ethanolic sulfuric acid (5% *v*/*v*). Flash column chromatography was performed on Merck silica gel (230–400 mesh, ASTM, West Conshohocken, PA, USA). Infrared spectra were recorded on a Bruker Alpha FTIR equipped with a Bruker universal ATR sampling accessory. ^1^H and ^13^C NMR spectra were recorded on Bruker Avance DPX300 and Bruker Avance 500 Ultrashield spectrometers equipped with QNP ^1^H/^13^C/^19^F/^31^P and inverse TBI ^1^H/^31^P/BB probes, respectively. Standard Bruker software was used for acquisition and processing routines. Chemical shifts are given in parts per million (ppm) and referenced to internal TMS (δ_H_, δ_C_ 0.00). *J* values are given in hertz (Hz). MALDI-TOF mass spectra were recorded on a 4800 Plus AB SCIEX spectrometer with 2,5-dihydroxybenzoic acid (DHB) as the matrix. ESI-TOF mass spectra were measured on a Waters Xevo Qtof spectrometer. All aqueous procedures used pure water (Milli-Q, 18.2 MΩ cm) obtained from a Millipore Milli-Q Plus system. Centrifugal filtrations were carried out on a Digicen 21R centrifuge using Amicon Millipore 10 kDa MWCO and 3 kDa MWCO centrifugal filters for purification and concentration purposes, respectively. Transmission electron microscopy (TEM) analyses were carried out on a Carl Zeiss LIBRA 120 PLUS instrument at 120 keV. All reagents were purchased from Sigma Aldrich and used without further purification. Compounds **2** [36], **3** [31,32], **15** [36], and **16** [36] were synthesized as previously reported. Human galectin-3 was expressed and purified as previously reported [45].

### 2.2. Synthesis

#### 2.2.1. 1-(Hydroxymethyl)-1′-({4-[4-O-(β-d-Galactopyranosyl)-β-d-glucopyranosyloxymethyl]-1H-1,2,3-tria-zol-1-yl} methyl)ferrocene (**5**)

CuSO_4_ (40 mg, 0.183 mmol) and sodium ascorbate (160 mg, 1.003 mmol) were added to a stirred solution of compounds **3** (400 mg, 1.476 mmol) and **4** (465 mg, 1.222 mmol) in THF/H_2_O (24 mL, 1:1) under N_2_. The resulting mixture was stirred at room temperature for 16 h and then −10% aqueous NH_3_ was added (20 mL). The resulting solution was filtered through silica gel. After that, the solvent was evaporated to dryness at reduced pressure avoiding heating over 40 °C. The crude was purified by column chromatography on silica gel (EtOAc/MeOH, 2:1) to obtain compound **5** (790 mg, 1.213 mmol, 99 %) as a yellow solid. IR (KBr, cm^−1^) 3373, 2925, 2882, 1665, 1401, 1380, 1331, 1236, 1156, 1116, 1051, 921, 893, 511, 486; ^1^H NMR (300 MHz; CD_3_OD) 7.97 (s, 1H, H-5-C2HN3), 5.22 (s, 2H, CH2N3), 4.96 (d, 1H, 2*J* = 12.4 Hz, CHO), 4.82 (d, 1H, 2*J* = 12.4 Hz, CHO), 4.44 (d, 1H, 3*J*_1,2_ = 7.6 Hz, H-1), 4.41−4.36 (m, 3H, H-1′, CpCH_2_OH), 4.34 (t, 2H, ^3^*J* = 1.7 Hz, H_Cp_), 4.28 (t, 2H, ^3^*J* = 1.7 Hz, H_Cp_), 4.23 (t, 2H, ^3^*J* = 1.7 Hz, H_Cp_), 4.21 (t, 2H, ^3^*J* = 1.7 Hz, HCp), 3.95 (d, 1H, ^2^*J*_6,6′_ = 11.4 Hz, H-6), 3.91−3.83 (m, 2H, H-6, 4′), 3.82−3.67 (m, 3H, H-6′, 6′, 5′), 3.66−3.51 (m, 3H, H-4, 3, 2′), 3.50−3.43 (m, 1H, H-3′), 3.40−3.37 (m, 1H, H-5), 3.32−3.27 (m, 1H, H-2); ^13^C NMR (75 MHz; CD_3_OD) 145.5 (C-4-C_2_HN_3_), 124.9 (C-5-C_2_HN_3_), 105.1 (C-1′), 103.3 (C-1), 89.2 (C_Cp_), 83.3 (C_Cp_), 80.6 (C-4), 77.0 (C-5′), 76.5 (C-5), 76.2 (C-3), 74.7 (C-3′), 74.6 (C-2), 72.5 (C-2′), 70.5 (C_Cp_), 70.4 (C_Cp_), 70.3 (C_Cp_), 70.2 (C-4′), 70.1 (C_Cp_), 63.0 (CH_2_O), 62.5 (C-6′), 61.9 (C-6), 60.9 (CH_2_OH), 50.9 (CH_2_N_3_); *m*/*z* (HRESI-TOF): Calc. for C_27_H_37_FeN_3_O_12_ 651.1727. Found: 652.1215 [M + H]^+^, 673.1620 [M + Na]^+^.

#### 2.2.2. 1-(Azidomethyl)-1′-({4-[4-O-(β-d-galactopyranosyl)-β-d-glucopyranosyloxymethyl]-1H-1,2,3-triazol-1-yl}methyl)ferrocene (**1**)

Compound **5** (650 mg, 0.998 mmol) was dissolved in an aqueous solution of NaN_3_ (1% *w*/*v*) acidified with concentrated HCl until pH 1 (−0.5% HCl). The resulting mixture was stirred at room temperature for 2 h. Then, it was neutralized with aqueous NaOH until pH 8 and lyophilized. The crude was purified by column chromatography on silica gel (EtOAc/MeOH, 2:1) to obtain compound **1** (607 mg, 0.898 mmol, 90%) as a yellow solid. IR (KBr, cm^−1^) 3373, 2924, 2885, 2099, 1816, 1645, 1378, 1328, 1259, 1238, 1225, 1156, 1116, 1058, 893, 834, 790, 705, 640, 507, 488; ^1^H NMR (300 MHz; CD_3_OD) 8.03 (s, 1H, H-5-C_2_HN_3_), 5.38 (s, 2H, CH_2_N_3_), 4.97 (d, 1H, ^2^*J* = 12.4 Hz, CHO), 4.79 (d, 1H, ^2^*J* = 12.4 Hz, CHO), 4.46 (d, 1H, ^3^*J*_1,2_ = 6.9 Hz, H-1), 4.43−4.37 (m, 3H, H-1′, H_Cp_), 4.32 (t, 2H, ^3^*J* = 1.6 Hz, HCp), 4.31−4.25 (m, 4H, H_Cps_), 4.18 (s, 2H, CH_2_N_3_), 4.12−3.72 (m, 5H, H-4′, 6, 6′), 3.70−3.46 (m, 6H, H-2, 3, 3′, 4, 5, 5’) 3.39−3.28 (m, 1H, H-2, CHD_2_OD); ^13^C NMR (75 MHz; CD_3_OD) 145.4 (C-4-C_2_HN_3_), 124.9 (C-5-C_2_HN_3_), 105.1 (C-1′), 103.2 (C-1), 84.4 (C_Cp_), 83.7 (C_Cp_), 80.6 (C-4), 77.0 (C-5′), 76.5 (C-5), 76.0 (C-3), 74.6 (C-2, 3′), 72.4 (C-2′), 70.8 (C_Cp_), 70.7 (C_Cps_), 70.6 (C_Cp_), 70.3 (C-4′), 63.0 (CH_2_O), 62.5 (C-6′), 61.7 (C-6), 51.49 (CH_2_–C_2_HN_3_), 50.8 (CH_2_N_3_); *m*/*z* (HRESI-TOF): Calc. for C_27_H_36_FeN_6_O_11_ 676.1791. Found: 652.1377 [M − N_2_]^+^, 699.1388 [M + Na]^+^.

#### 2.2.3. General Procedure for the Synthesis of Alkyne-Terminated PAMAM Dendrimers **9–11**

Commercially available 20 wt % solutions of amine-terminated PAMAM dendrimers **6**–**8** in methanol were mixed with an equal volume of a solution of CH_2_Cl_2_ containing pent-4-ynoic anhydride (3 eq. per amino group). The resulting mixture was stirred at room temperature for 3 days. Diethyl ether was added until a white precipitate appeared. The solid was filtered off, rigorously washed with plenty of diethyl, and used in the next step without further purification.

##### G0-PAMAM-(COCH_2_CH_2_C≡CH)_4_ (**9**)

Starting from a 20 wt % solution of **6** (85 µL, 0.028 mmol) in methanol, the procedure yielded **9** as a white solid (19 mg, 0.023 mmol, 81%). IR (KBr, cm^−1^): 3288, 3085, 2941, 2835, 1635, 1552, 1442, 1374, 1298, 1272, 1240, 1126, 1099, 1016, 960, 885, 731, 687, 639; ^1^H NMR (300 MHz, DMSO-*d*_6_): 7.94 (bs, 8H, NH), 3.07 (bs, 16H, CH_2_NHCO), 2.76 (t, 4H, ^4^*J* = 2.5 Hz, HC≡C), 2.63 (t, 8H, ^3^*J* = 6.8 Hz, CH2N), 2.42 (bs, 4H, CH_2_N), 2.38−2.31 (m, 8H, CH_2_C≡CH), 2.29−2.22 (m, 8H, CH_2_CO), 2.31 (t, 8H, ^3^*J* = 6.8 Hz, CH_2_CO); ^13^C NMR (75 MHz, DMSO-*d*_6_): 171.5 (CO), 170.4 (CO), 83.7 (C≡CH), 71.3 (C≡CH), 50.9 (CH2N), 49.6 (CH2N), 38.4 (CH_2_NHCO), 38.3 (CH_2_NHCO), 34.2 (CH_2_CO), 33.2 (CH_2_CO), 14.2 (CH_2_C≡CH); *m*/*z* (ESI-TOF): Calc. for C_42_H_64_N_10_O_8_ 836.5. Found: 841.5 [M + 5H]^+^.

##### G1-PAMAM-(COCH_2_CH_2_C≡CH)_8_ (**10**)

Starting from a 20 wt % solution of **7** (204 µL, 0.023 mmol) in methanol, the procedure yielded **10** as a white solid (37 mg, 0.018 mmol, 78%). IR (KBr, cm^−1^): 3279, 3081, 2936, 2835, 2117, 1635, 1537, 1430, 1362, 1234, 1164, 1125, 1037, 644; ^1^H NMR (300 MHz, DMSO-*d*_6_): 8.21−7.65 (bs, 20H, NH), 3.08 (bs, 40H, CH_2_NHCO), 2.74 (bs, 8H, HC≡C), 2.64 (bs, 24H, CH_2_N), 2.45−2.14 (m, 68H, CH_2_N, CH_2_C ≡ CH, CH_2_CO); ^13^C NMR (75 MHz, DMSO-*d*_6_): 172.0 (CO), 170.9 (CO), 84.1 (C≡C), 71.7 (C≡CH), 52.5 (CH2N), 49.9 (CH_2_N), 38.8 (CH_2_NHCO), 38.7 (CH_2_NHCO), 37.3 (CH_2_NHCO), 34.9 (CH_2_CO), 34.6 (CH_2_CO), 33.6 (CH_2_CO), 14.6 (CH_2_C≡CH); *m*/*z* (ESI-TOF): Calc. for C_102_H_160_N_26_O_20_ 2070.2. Found: 2071.2 [M + H]^+^.

##### G2-PAMAM-(COCH_2_CH_2_C≡CH)_16_ (**11**)

Starting from a 20 wt % solution of **8** (221 µL, 0.012 mmol) in methanol, the procedure yielded **11** as a white solid (43 mg, 0.009 mmol, 79%). IR (KBr, cm^−1^): 3286, 3084, 2940, 2835, 1633, 1540, 1432, 1368, 1238, 1167, 1125, 1023, 638; ^1^H NMR (300 MHz, DMSO-*d*_6_): 8.39−7.58 (bs, 44H, NH), 3.08 (bs, 88H, CH_2_NHCO), 2.80−2.59 (bs, 72H, HC≡C, CH2N), 2.46−2.04 (m, 148H, CH_2_N, CH_2_ ≡CH, CH_2_CO); ^13^C NMR (75 MHz, DMSO-*d*_6_): 172.0 (CO), 171.7 (CO), 170.9 (CO), 84.1 (C≡CH), 71.7 (C≡CH), 52.5 (CH_2_N), 49.9 (CH_2_N), 38.8 (CH_2_NHCO), 38.7 (CH_2_NHCO), 37.3 (CH_2_NHCO), 34.6 (CH_2_CO), 33.6 (CH_2_CO), 14.6 (CH_2_C≡CH); *m*/*z* (ESI-TOF): Calc. for C_222_H_353_N_58_O_44_ 4536.7157. Found: 4538.7 [M + 4H]^+^.

#### 2.2.4. General Procedure for the Synthesis of Electroactive Lactosylated PAMAM Dendrimers **12**–**14**

The alkyne-terminated PAMAM dendrimers (**9**–**11**) and **1** (1.5 eq. per alkyne group) were dissolved in THF/H_2_O 1:1 (0.04 mL per mg of **5**) under N_2_ atmosphere. Then, CuSO_4_ (0.2 eq. per alkyne group) and sodium ascorbate (0.4 eq. per alkyne group) were added to the solution in this order. The resulting mixture was stirred at room temperature for 20 h. After that, THF was evaporated under vacuum without heating over 40 °C. The resulting solution was diluted until −10 mL with 5% aqueous NH_3_ and the product was isolated by repetitive centrifugal filtration (3 kDa cut-off, 10× dilution) with 5 consecutive concentration and re-dissolution steps. The final solution was lyophilized to obtain the corresponding electroactive lactosylated PAMAM dendrimer as a yellow solid.

##### G0-PAMAM-(COCH_2_CH_2_TACH_2_FcCH_2_TACH_2_Lac)_4_ (**12**)

Starting from **9** (21 mg, 0.025 mmol) in methanol, the procedure yielded **12** as a yellow solid (64 mg, 0.018 mmol, 72%). IR (KBr, cm^−1^): 3412, 2930, 1646, 1553, 1435, 1383, 1338, 1237, 1126, 1058, 815, 712, 623, 508, 493; ^1^H NMR (300 MHz, DMSO-*d*_6_): 8.06 (s, 4H, H5-C_2_HN_3_), 8.00−7.85 (m, 8H, NH), 7.76 (s, 4H, H5′-C_2_HN_3_), 5.32 (s, 8H, CH_2_N_3_), 5.27 (s, 8H, CH_2_N_3_), 5.20−5.05 (m, 8H, OHs), 4.83 (d, 4H, ^2^*J* = 12.4 Hz, CHO), 4.82−4.76 (m, 8H, OHs), 4.73−4.60 (m, 12H, OHs), 4.61 (d, 4H, ^2^*J* = 12.4 Hz, CHO), 4.39−4.29 (m, 20H, H_Cp_, H-1′), 4.23−4.16 (m, 20H, H_Cp_, H-1), 3.80 (dd, 4H, ^2^*J*_6’,6_ = 11.2 Hz, ^3^*J*_6’,5_ = 11.2 Hz, H-6′), 3.65−3.40 (m, 16H, H-4′,5, 5′,6), 3.39−3.18 (m, H-2, 2′, 3, 3′, 4, HDO), 3.05 (bs, 16H, CH_2_NHCO), 2.81 (t, 8H, ^3^*J* = 7.6 Hz, CH_2_-C_2_HN_3_), 2.63 (bs, 8H, CH_2_N), 2.45–2.33 (m, 12H, CH_2_N, CH_2_CO), 2.19 (bs, 8H, CH_2_CO); ^13^C NMR (75 MHz, DMSO-*d*_6_): 171.5 (CO), 171.3 (CO), 146.0 (C4-C_2_HN_3_), 143.7 (C4-C_2_HN_3_), 123.7 (C5-C_2_HN_3_), 121.4 (C5-C_2_HN_3_), 103.8 (C-1’), 101.8 (C-1), 83.2 (C_Cp_), 82.9 (C_Cp_), 80.7 (C-4), 75.5 (C-5’), 74.9 (C-3, 5), 73.2 (C-2′), 73.0 (C-2), 70.5 (C-3′), 69.6 (C_Cp_), 69.5 (C_Cp_), 69.3 (C_Cp_), 69.2 (C_Cp_), 68.1 (C-4′), 61.7 (CH_2_O), 60.5 (C-6), 60.4 (C-6′), 50.8 (CH_2_N), 49.6(CH_2_N), 48.6 (CH_2_N_3_), 48.5 (CH_2_N_3_), 38.3 (CH2NHCO), 34.9 (CH_2_CO), 33.2 (CH_2_CO), 21.2 (CH_2_-C_2_HN_3_); *m*/*z* (HRESI-TOF): Calc. for C_150_H_208_Fe_4_N_34_O_52_ 3542.2108. Found: 1772.1643 [M + 2H]^2+^, 1181.3887 [M + 3H]^3+^, 886.2606 [M + 4H]^+^. Number of expected lactose units per dendrimer: 4. Found by sulfuric acid–phenol assays: 4.10 ± 0.21 (see the Supplementary Materials).

##### G1-PAMAM-(COCH_2_CH_2_TACH_2_FcCH_2_TACH_2_Lac)_8_ (**13**)

Starting from **10** (60 mg, 0.029 mmol) in methanol, the procedure yielded **13** as a yellow solid (155 mg, 0.021 mmol, 73%). IR (KBr, cm^−1^): 3363, 2956, 2922, 2852, 1731, 1653, 1553, 1461, 1435, 1378, 1339, 1237, 1129, 1057, 973, 816, 684, 642, 510, 489; ^1^H NMR (300 MHz, DMSO-*d*_6_): 8.06 (s, 8H, H5-C_2_HN_3_), 8.00–7.81 (m, 20H, NH), 7.76 (s, 8H, H5′-C_2_HN_3_), 5.31 (s, 16H, CH_2_N_3_), 5.27 (s, 16H, CH_2_N_3_), 5.19 (bs, 8H, OHs), 5.11 (bs, 8H, OHs), 4.89−4.76 (m, 16H, CHO, OH), 4.74−4.48 (m, 40H, CHO, OHs), 4.39−4.27 (m, 40H, H_Cp_, H-1′), 4.26−4.15 (m, 40H, H_Cp_, H-1), 3.84−3.74 (m, 8H, H-6′), 3.65−3.34 (m, 64H, H-4′, 5, 5′, 6, 2, 2′, 3, 3′, 4), 3.06 (bs, 40H, CH_2_NHCO), 2.81 (t, 16H, ^3^*J* = 7.0 Hz, CH_2_-C_2_HN_3_), 2.64 (bs, 24H, CH_2_N), 2.45−2.33 (m, 28H, CH_2_N, CH_2_CO), 2.18 (bs, 24H, CH_2_CO); ^13^C NMR (75 MHz, DMSO-*d*_6_): 171.6 (CO), 171.4 (CO), 171.3 (CO), 146.0 (C4-C_2_HN_3_), 143.7 (C4-C_2_HN_3_), 123.7 (C5-C_2_HN_3_), 121.4 (C5-C_2_HN_3_), 103.9 (C-1’), 101.9 (C-1), 83.1 (C_Cp_), 82.8 (C_Cp_), 80.7 (C-4), 75.5 (C-5′), 74.9 (C-3, 5), 73.2 (C-2′), 73.0 (C-2), 70.5 (C-3’), 69.6 (C_Cps_), 69.3 (C_Cp_), 69.2 (C_Cp_), 68.1 (C-4′), 61.7 (CH_2_O), 60.5 (C-6), 60.4 (C-6′), 52.2 (CH_2_N), 49.5(CH_2_N), 48.6 (CH_2_N_3_), 48.5 (CH_2_N_3_), 38.4 (CH_2_NHCO), 38.3 (CH_2_NHCO), 36.9 (CH_2_NHCO), 34.9 (CH_2_CO), 33.2 (CH_2_CO), 21.2 (CH_2_-C_2_HN_3_); *m*/*z* (MALDI-TOF): Calc. for C_318_H_448_Fe_8_N_74_O_108_ 7481.7 (EM); Found: 7481.3 [M]^+^. Number of expected lactose units per dendrimer: 8. Found by sulfuric acid–phenol assays: 7.97 ± 0.23 (see Supplementary Materials).

##### G2-PAMAM-(COCH_2_CH_2_TACH_2_FcCH_2_TACH_2_Lac)_16_ (**14**)

Starting from **11** (40 mg, 0.009 mmol) in methanol, the procedure yielded **14** as a yellow solid (101 mg, 0.007 mmol, 73%). IR (KBr, cm^−1^): 3415, 2931, 1643, 1552, 1436, 1373, 1337, 1327, 1128, 1056, 813, 716, 682, 591, 493; ^1^H NMR (300 MHz, DMSO-*d*_6_): 8.06 (s, 16H, H5-C_2_HN_3_), 8.00−7.77 (m, 44H, NH), 7.76 (s, 16H, H5′-C_2_HN_3_), 5.31 (s, 32H, CH_2_N_3_), 5.27 (s, 32H, CH_2_N_3_), 5.20 (bs, 16H, OHs), 5.11 (bs, 16H, OHs), 4.89−4.75 (m, 32H, CHO, OH), 4.74−4.47 (m, 80H, CHO, OHs), 4.39−4.28 (m, 80H, H_Cp_, H-1′), 4.26−4.10 (m, 80H, H_Cp_, H-1), 3.87−3.74 (m, 16H, H-6’), 3.70−3.41 (m, 64H, H-4′,5, 5′,6), 3.40−3.21 (m, H-2, 2′, 3, 3′, 4, HDO), 3.06 (bs, 84H, CH_2_NHCO), 2.81 (t, 32H, ^3^*J* = 7.3 Hz, CH_2_-C_2_HN_3_), 2.65 (bs, 56H, CH2N), 2.48−2.31 (m, 60H, CH_2_N, CH_2_CO), 2.18 (bs, 56H, CH_2_CO); ^13^C NMR (75 MHz, DMSO-*d*_6_): 171.6 (CO), 171.4 (CO), 171.3 (CO), 146.0 (C4-C_2_HN_3_), 143.7 (C4-C_2_HN_3_), 123.7 (C5-C_2_HN_3_), 121.4 (C5-C_2_HN_3_), 103.9 (C-1’), 101.9 (C-1), 83.1 (C_Cp_), 82.9 (C_Cp_), 80.7 (C-4), 75.5 (C-5′), 74.9 (C-3, 5), 73.2 (C-2’), 73.0 (C-2), 70.5 (C-3′), 69.7 (C_Cp_), 69.6 (C_Cp_), 69.4 (C_Cp_), 69.3 (C_Cp_), 68.2 (C-4′), 61.7 (CH_2_O), 60.5 (C-6), 60.4 (C-6′), 52.1 (CH_2_N), 49.5(CH_2_N), 48.7 (CH_2_N_3_), 48.5 (CH_2_N_3_), 38.4 (CH2NHCO), 38.3 (CH_2_NHCO), 36.9 (CH_2_NHCO), 34.9 (CH_2_CO), 33.2 (CH_2_CO), 21.2 (CH_2_-C_2_HN_3_); *m*/*z* (ESI-TOF): Calc. for C_654_H_928_Fe_16_N_154_O_220_ 15361 Found: 15361 [M]^+^. Number of expected lactose units per dendrimer: 16. Found by sulfuric acid–phenol assays: 16.35 ± 1.11 (see Supplementary Materials).

#### 2.2.5. Bis [2 3-(3-{1-[1′-({4-[4-O-(β-d-galactopyranosyl)-β-d-glucopyranosyloxymethyl]-1H-1,2,3-triazol-1-yl}methyl)ferrocenylmethyl]-1H-1,2,3-triazol-4-yl}propanoylamino)-12,15,18,21-tetraoxatricosanyl] disulfide (**17**)

CuSO_4_ (7 mg, 0.032 mmol) and sodium ascorbate (10 mg, 0.063 mmol) were added to a stirred solution of compounds **16** (70 mg, 0.076 mmol) and **1** (113 mg, 0.167 mmol) in THF/H_2_O (6 mL, 1:1) under N_2_. The resulting mixture was stirred at room temperature overnight and then the solvent was evaporated to dryness at reduced pressure. The crude was purified by column chromatography on silica gel (CH_3_CN/H_2_O/NH_3(aq_._)_ 30%, 8:2:1 → 10:4) to obtain compound **17** (164 mg, 0.072 mmol, 95%) as a yellow solid. IR (KBr, cm^−1^): 3390, 2931, 1635, 1237, 1167, 1126, 1097, 1079, 1061, 1025, 840, 815, 583, 501 cm^−1^; ^1^H NMR (300 MHz, DMSO-*d*_6_): 8.08 (s, 2H, H-5-C_2_HN_3_), 7.92 (bs, 2H, NHs), 7.75 (s, 2H, H-5-C_2_HN_3_), 5.31 (s, 4H, CH_2_-C_2_HN_3_), 5.26 (s, 4H, CH_2_-C_2_HN_3_), 4.82 (d, ^2^*J* = 12.0 Hz, 2H, CHO), 4.60 (d, ^2^*J* = 12.0 Hz, 2H, CHO), 4.40−4.27 (m, 6H, H_Cp_, H-1), 4.24−4.14 (m, 6H, H_Cp_, H-1′), 3.86−3.74 (m, 6H, H-2, 6), 3.67−3.13 (m, H-2′, 3, 3′, 4, 4′, 5,5′,6′, CH_2_OEG, *CH_2_*NHCO, O*CH_2_*(CH_2_)_10_, HDO), 3.01 (t, ^3^*J* = 7.5 Hz, 4H, *CH_2_*CONH), 2.80 (t, ^3^*J* = 7.0 Hz, 4H, *CH_2_*-C_2_HN_3_), 2.67 (t, ^3^*J* = 7.1 Hz, 4H, CH_2_S), 1.67−1.53 (m, 4H, (CH_2_)_9_), 1.52−1.39 (m, 4H, (CH_2_)_9_), 1.38−1.12 (m, 28H, (CH_2_)_9_); ^13^C NMR (75 MHz, DMSO-*d*_6_): 171.6 (CO), 146.0 (C4-C_2_HN_3_), 143.7 (C4-C_2_HN_3_), 123.7 (C5-C_2_HN_3_), 121.4 (C5-C_2_HN_3_), 103.8 (C-1′), 101.8 (C-1), 83.2 (C_Cp_), 82.9 (C_Cp_), 80.7 (C-4), 75.5 (C-5′), 74.9 (C-3, 5), 73.2 (C-2′), 73.1 (C-2), 70.5 (C-3′), 70.3 (CH_2_OEG), 69.8 (CH_2_OEG), 69.7 (CH_2_OEG), 69.6 (C_Cp_), 69.5 (C_Cp_), 69.4 (CH_2_OEG), 69.3 (C_Cp_), 69.2 (C_Cp_), 69.1 (CH_2_OEG), 68.1 (C-4′), 61.7 (CH_2_O), 60.5 (C-6), 60.4 (C-6′), 48.6 (CH_2_N_3_), 48.4 (CH_2_N_3_), 38.5 (*CH_2_*NHCO), 37.9 (CH_2_S), 34.7 (*CH_2_*CONH), 29.2, 29.0, 28.9, 28.5, 25.6 [(CH_2_)_9_], 21.2 (CH_2_-C_2_HN_3_); *m*/*z* (ESI-TOF): Calc. for C_102_H_160_Fe_2_N_14_O_32_S_2_ 2269.9497 Found: 2270.5857 [M + H]^+^.

#### 2.2.6. Synthesis of Citrate-Stabilized AuNPs

Citrate-stabilized AuNPs were prepared according to a modification [46] of the Turkevich protocol [47]. Briefly, a degassed solution of sodium citrate (228 mg, 0.775 mmol) in Milli-Q water (20 mL) at 55 °C was added to a refluxing degassed solution of HAuCl_4_·3H_2_O (79 mg, 0.201 mmol) in Milli-Q water (200 mL) under vigorous stirring. The mixture was refluxed for 30 min and cooled to room temperature before being filtered through a Sartorius Minisart 0.2 μm filter to provide citrate-stabilized AuNPs of 12.1 ± 1.0 nm of average diameter in a concentration of 16 nM, as determined by UV–vis spectroscopy [48] and TEM. The citrate-stabilized AuNPs solution was washed with 1 mM NaOH [36,49] by repetitive centrifugal filtration (10 kDa cut-off, 10× dilution) with three consecutive concentration and re-dissolution steps to obtain 16 nM AuNPs solution in 1 mM NaOH with an average size of 12.1 ± 1.2 nm as determined by TEM.

#### 2.2.7. Preparation of AuNP@Fc-Lac

A 2.5 mM solution of disulfide **17** in methanol (2 mL) was added to a stirred solution of 16 nM citrate-stabilized AuNPs in 1 mM NaOH (10 mL) and the resulting solution was stirred for 24 h. After that, THF (10 mL) was added and the resulting suspension was precipitated by centrifugation at 4000 rpm for 20 min. The supernatant was removed and the functionalized AuNPs were re-dissolved in H_2_O (10 mL). THF (10 mL) was added and the resulting suspension was precipitated again by centrifugation at 4000 rpm for 20 min. The process was repeated 3 times. After that, the functionalized AuNPs were dissolved in 10 mm phosphate buffer (pH 7.2) and dialyzed by centrifugal filtration (10 x dilution, Millipore Amicon Centriplus 10 kDa). The process was repeated 4 times to obtain a final solution of 20 nm AuNP@Fc-Lac (8 mL) in 10 mm phosphate buffer (pH 7.2). Average diameter of the gold core = 12.2 ± 1.1 nm (from TEM analysis). Number of ligands/AuNP = 1659 ± 94 (from elemental gold-to-iron ratio as determined by ICP-OES). Concentrations were determined by transmission electron microscopy (TEM) and UV–visible spectroscopy [48].

### 2.3. UV–Visible Experiments

UV–visible measurements were performed using Jasco V-530 spectrophotometer at room temperature using a 1 cm path length. For protein assays, aqueous dispersions of AuNP@Fc-Lac (2 nM) in phosphate buffer 10 mM at pH 7.2 with 20 mM NaCl were treated with a final concentration of 10.0 µM of Gal-3 or bovine serum albumin.

### 2.4. Voltammetric Experiments

**Figure 1:**
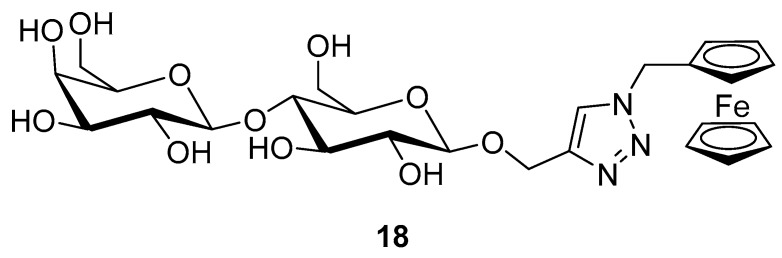
Chemical structure of compound **18**.

Electrochemical measurements were carried out in sonicated, nitrogen-purged aqueous (Milli-Q 18.2 MΩ cm) solution with a micro-Autolab type III connected to a personal computer running Eco Chimie B. V. GPES 4.9 software. The electrodes were carefully cleaned before each experiment. The glassy carbon disk working electrode (Ø 2 mm, effective area 0.038 ± 0.006 cm^2^) was immersed in a 0.1 M HNO_3_ solution for 5 min and polished with a basic Al_2_O_3_ water slurry. The platinum sheet counter electrode (6 × 4 mm, effective area 0.410 ± 0.003 cm^2^) was immersed in a 50% *v*/*v* H_2_SO_4_ solution for 5 min. Both electrodes were then sonicated in a 1:1:1 H_2_O/MeOH/CH_3_CN mixture for 5 min prior to use. The effective area of the electrodes was determined as previously reported [39]. An Ag/AgCl (3 M KCl) electrode was used as a reference. Solutions of conjugate **18** (Figure 1) and dendrimers **12**–**14** (50 µM for **18**, 25 µM for **12**, 12.5 µM for **13** and 6.25 µM for **14**) and increasing amounts of Gal-3 varying from 0 to 45 µM were prepared in 10 mM phosphate buffer at pH 7.2 with 20 mM NaCl and incubated for 10 min at room temperature. Before each measurement with AuNP@Fc-Lac, the glassy carbon electrode was modified by incubation in solutions of AuNP@Fc-Lac (20 nM) in 10 mM phosphate buffer at pH 7.2 with 20 mM NaCl for 1 h at room temperature and then washed with plenty of H_2_O. After that, it was allowed to equilibrate for 10 min in solutions of different concentrations of Gal-3 ranging from 0 to 500 nM. Nitrogen was bubbled through the solutions for 3 min before each measurement. Differential pulse voltammetry experiments were measured with a scan rate of 5 mVs^−1^, a step potential of 20 mV, a modulation amplitude of 50 mV, a modulation time of 0.05 s, and an interval time of 2 s between 0 and 1 V for AuNP@Fc-Lac, 0 and 0.8 V for dendrimers **12**–**14**, and 0 and 0.7 for **18**.

### 2.5. Isothermal Titration Calorimetry Measurements

Calorimetric experiments were conducted using either an MCS or an ultrasensitive VP-ITC (Microcal Inc., Northampton, MA, USA). The preparation of samples and isothermal titration calorimetry (ITC) experiments were carried out as previously described elsewhere [50]. Titrations were routinely performed in a 20 mM phosphate buffer at pH 7.2 with 150 mM NaCl at 25 °C. During titrations, the reaction mixture was continuously stirred at 370 rpm and the reference cell was filled with Milli-Q water. Blank titrations of ligand into buffer were also performed to correct the heat generated by dilution and mixing. The amount of heat produced per injection was calculated by integration of the individual peaks by the Origin software provided with the instrument. An equal and independent site model was used to fit the experimental data. This model provided the binding constant, the enthalpy change, and the number of binding sites (n) along with the corresponding standard deviations. The changes in the standard free energy Δ*G*^0^ and entropy Δ*S*^0^ were determined as Δ*G*^0^ = −R*T*ln*K* and TΔ*S*^0^ = Δ*H* − Δ*G*^0^ (assuming that Δ*H* = Δ*H*^0^).

## 3. Results and Discussion

### 3.1. Synthesis

**Scheme 1:**
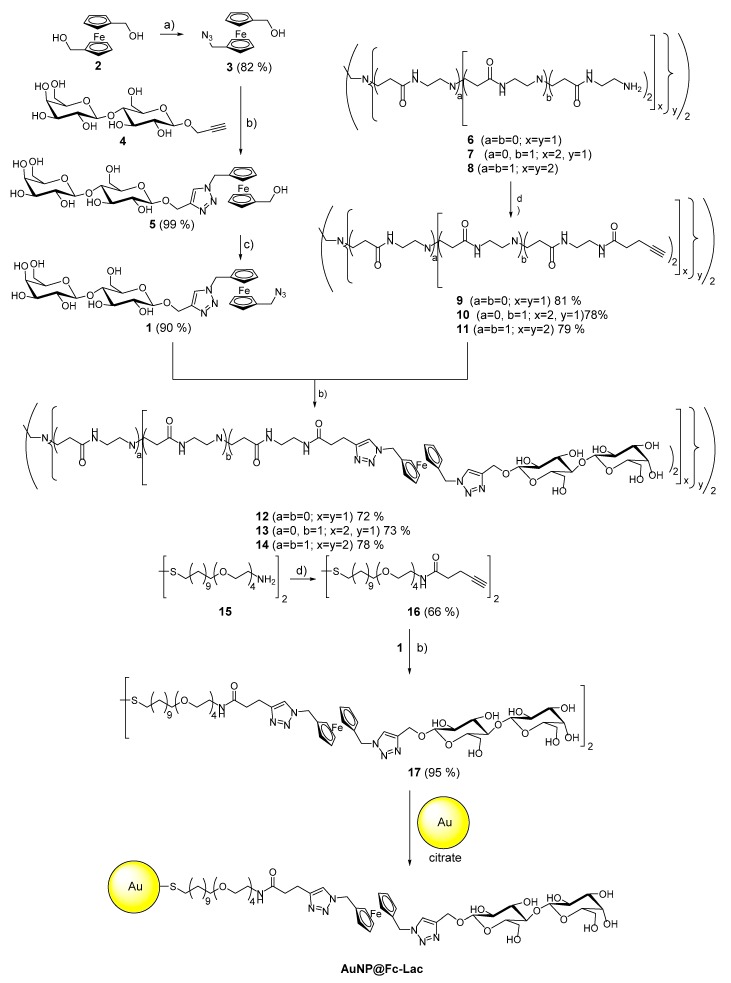
(**a**) NaN_3_, HCl_._, EtOAc/H_2_O, room temperature, 45 min; (**b**) CuSO_4_, sodium ascorbate, THF/H_2_O, room temperature, overnight; (**c**) NaN_3_, HCl_(aq)_, room temperature, 2 h; (**d**) pent-4-ynoic anhydride, CH_2_Cl_2_/MeOH, room temperature, overnight.

To generate multivalent presentations of ferrocene–lactose (Fc-Lac) conjugates, we chose PAMAM dendrimers and Au nanoparticles (AuNPs) as scaffolds for the display of the conjugates. We started our studies with the preparation of building block **1**, consisting of an azide-functionalized lactose–ferrocene conjugate, which is ready for further conjugation or direct coupling using azide-alkyne cycloaddition (AAC). This building block **1** was key for the convenient synthesis of the multivalent electrochemical probes. Commercially available 1,1′-bis(hydroxymethyl)ferrocene **2** was regioselectively transformed, in one step, into mono-azidoferrocene **3** in high a yield (Scheme 1) [36]. Cu(I)-catalyzed AAC of ferrocene derivative **3** with propargyl β-d-lactoside **4** [45] afforded Fc-Lac conjugate **5** in an almost quantitative yield. The treatment of such conjugate with sodium azide in aqueous HCl allowed the introduction of a new azido group in a high yield. The resulting azido-functionalized Fc-Lac **1** was then used for an additional Cu (I)-catalyzed AAC with alkyne-terminated PAMAM dendrimers **9**–**11** to obtain Fc-Lac dendrimers **12**–**14** in 78–81% yields. Similarly, azido-functionalized Fc-Lac **1** reacted with alkynylated disulfide **16** [36] to obtain Fc-Lac disulfide **17** in a 95% yield. Alkyne-terminated PAMAM dendrimers **9**–**11** and disulfide **16** were prepared via the reaction of the corresponding amino derivatives **6**–**8** and **15** with pent-4-ynoic anhydride [51] (Scheme 1).

The ^1^H- and ^13^C-NMR analysis of Fc-Lac dendrimers **12**–**14** and disulfide **17** evidenced the presence of two chemically different 1,2,3-triazole rings. The ^1^H NMR spectra revealed two singlets at 8.06–8.08 and 7.75–7.76 ppm, corresponding to the 1,2,3-triazole methine protons. The ^13^C NMR spectra displayed four signals: two at 146.0 and 143.7 ppm and two at 123.7 and 121.4, corresponding to the triazole C-4 and C-5 atoms, respectively. The ^13^C NMR spectra also showed two signals at 103.8–103.9 and 101.8–101.9 ppm corresponding to the anomeric carbons of the β-d-galacto- and β-d-glucopyranoside units of the β-d-lactoside moieties. In addition, the protons of the methylenes linking the triazole moieties to the metallocene units were clearly observable in the ^1^H NMR spectra as two singlets at 5.32–5.32 and 5.27–5.26 ppm. The ESI- and MALDI-TOF spectra confirmed the molecular weight of Fc-Lac dendrimers **12**–**14** and disulfide **17**. For the Fc-Lac dendrimers, the number of carbohydrate units per dendrimer was determined by means of sulfuric acid–phenol assays to be 4.1 ± 0.2, 8.0 ± 0.2 and 16.4 ± 1.1 for **12**, **13**, and **14**, respectively (see Supplementary Materials) [38].

Fc-Lac disulfide **17** was employed to functionalize citrated-capped AuNPs. Citrate-capped gold nanoparticles were prepared according to a modification [46] of the Turkevich protocol [47], as reported elsewhere [36,45,52]. Functionalized AuNPs were prepared by mixing an excess of Fc-Lac disulfide **17** with 12 nm citrate-capped AuNPs in 1 mM NaOH for 24 h at room temperature. After removing the unreacted disulfide **17**, the UV/Vis spectra of the resulting Fc-Lac-functionalized AuNPs (AuNPs@Fc-Lac) revealed a bathochromic shift of 8 nm of the SPR band. Redshift is a distinct indication of sulfur anchoring and has been attributed to changes in the electronic environment on the surface of AuNPs [36,45,53,54]. No broadening in the SPR band was observed, which excluded ligand-induced AuNPs aggregation. The average diameter (12.2 nm) of the nanoparticle gold cores was unaltered after functionalization, as determined by TEM analyses (see Supplementary Materials for details). The average number of ligands per nanoparticle was estimated to be 1765 ± 32 based on the elemental gold-to-iron ratio obtained by inductively coupled plasma optical emission spectroscopy (ICP-OES) in combination with the TEM data (see Supplementary Materials)

### 3.2. Electrochemical Characterization

**Figure 2:**
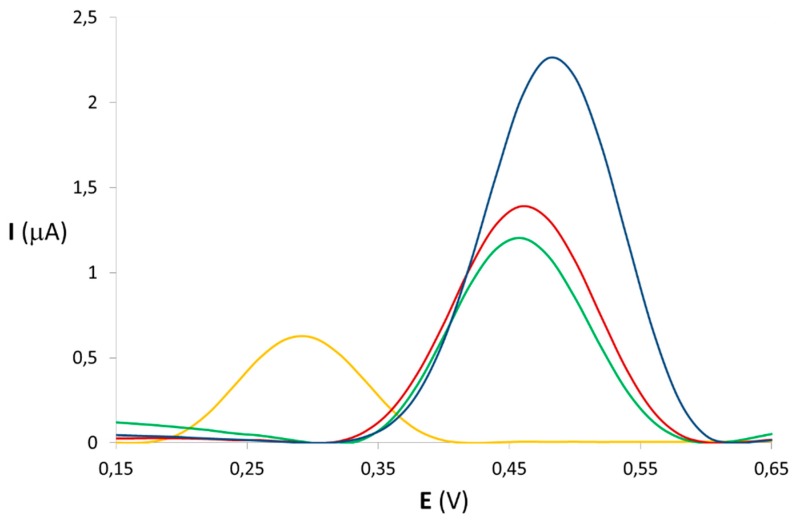
Differential pulse voltammograms for **12** (25 μM, green), **13** (12.5 μM, red), **14** (6.25 μM, blue), and **18** (50 μM, orange) in 10 mM phosphate buffer at pH 7.2 and 25 °C with 20 mM NaCl as the supporting electrolyte.

The differential pulse voltammograms of the Fc-Lac dendrimers **12**–**14** and mono-Fc-Lac conjugate 18 [32] in 10 mM phosphate buffer at pH 7.2 with 20 mM NaCl as the supporting electrolyte are shown in Figure 2. The voltammograms of multivalent compounds **12**–**14** showed only one peak potential (*E*_p_) (+0.456, +0.461, and +0.482 V, respectively) for the oxidation of the ferrocene moieties (Table 1), which is consistent with the apparent equivalence of all ferrocene residues displayed by each glycodendrimer at the timescale of the electrochemical measurement [38,55]. Contrary to the previously investigated mannosylferrocenyl PAMAM dendrimers [38], in which the observed oxidation potential was independent of the dendrimer generation, we observed a slight shift to a more positive *E*_p_ value of −0.026 V when going from dendrimer generation G0 to G2. This behavior may be related to the lesser polar microenvironment around the ferrocene residue with an increase of dendrimer generation. Moving from a monosaccharide to a disaccharide may lead to a more shielded ferrocene from solvent interactions and will, therefore, make the ferrocene moiety more difficult to oxidize. This shielding effect would be more effective in dendrimer **14** with the highest generation. Another variation from the previously reported glycoferrocenyl dendrimer is the substitution of an electron-withdrawing amide group directly attached to the ferrocenyl group by an electron-donating methyltriazole group, which led to lower *E*_p_ values than those reported (+0.560 V).

Interestingly, the DPV peak current (*I*_p_) values 0.62, 1.21, 1.39, and 2.26 µA for mono- **18**, tetra- **12**, octa- **13,** and hexadecamer **14**, respectively, increase with the glycodendrimer generation (Table 1). This trend is the opposite of that observed for the previously reported mannosylferrocenyl dendrimer [38]. The *I*_p_ value is directly proportional to the concentration and the square of the diffusion coefficient of the electroactive species, the number of exchanged electrons, and the electrode surface [56]. Hence, for lactosylferrocenyl dendrimers, the increase of the electrochemical signal that results from the increased number of exchanged electrons prevails over the expected decrease of the peak current due to the slower diffusion of the larger electroactive species. This behavior might be due to stronger adsorption of the larger dendrimer onto the electrode, which results in an enhancement of the *I*_p_ value [55].

The electrochemical properties of AuNP@Fc-Lac were also studied by DPV. DPV measurements of AuNP@Fc-Lac were carried out on a 20 nM nanoparticle solution in 10 mM phosphate buffer at pH 7.2 with 20 mM NaCl, after being incubated for 1 h in the presence of a glassy carbon working electrode, an Ag/AgCl (3 M KCl) reference electrode, and a Pt sheet counter electrode. The differential pulse voltammogram of AuNP@Fc-Lac showed an oxidation peak with a peak current value of 6.11 μA at an oxidation potential value of 0.620 V.

### 3.3. Binding Abilities Toward Galectin-3

We investigated the binding abilities of mono- **18**, tetra- **12**, octa- **13**, and hexadeca-Fc-Lac **14** toward Gal-3, as we envisaged that enhanced Gal-3 binding due the multivalent effect would contribute to improved sensing abilities.

**Figure 3:**
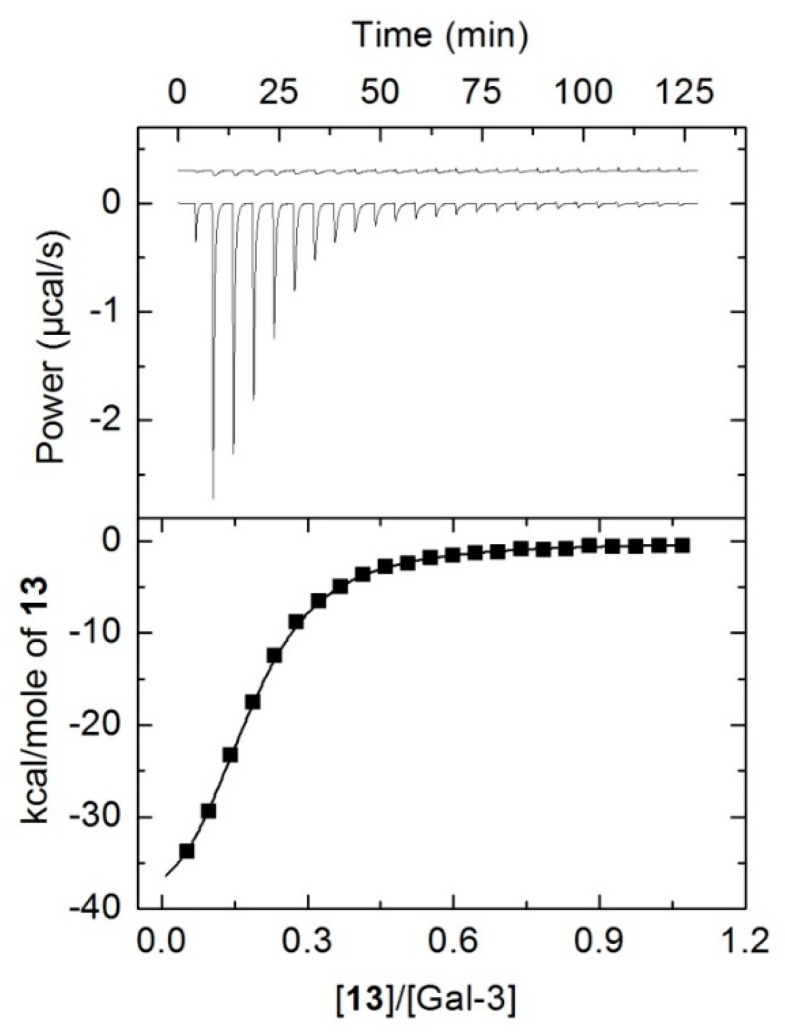
Representative titration of galectin-3 (Gal-3) (40.58 μM) with compound **13** (0.514 mM) in 20 mM phosphate buffer at pH 7.2 with 150 mM NaCl at 25 °C. The top panel shows the raw calorimetric data for 25 automatic injections, 5 µL each (1 µL for the first) of **13**. The area under each peak represents the amount of heat released upon binding of the conjugate to the lectin. The smooth solid lines (**bottom panel**) represent the best fit of the experimental data to a model of *n* equal and independent sites.

**Figure 4:**
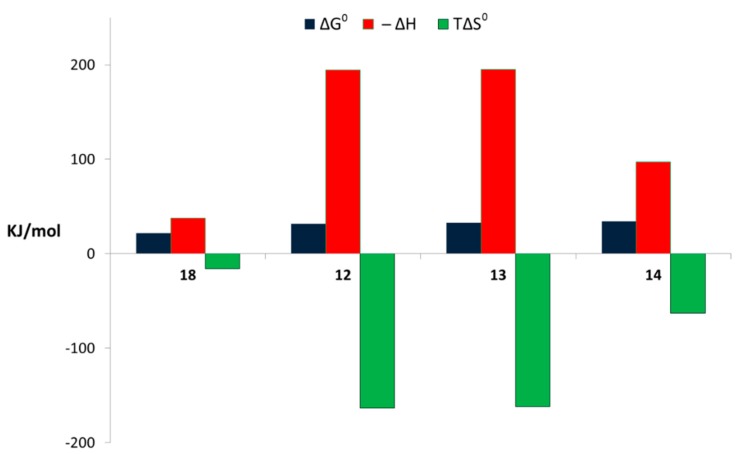
Free energy (−Δ*G*^0^, black), enthalpy (−Δ*H*, red), and entropy changes (*T*Δ*S*^0^, green) for the binding of Fc-Lac dendrimers **12**–**14** and monovalent glycoconjugate **18** to Gal-3 obtained from isothermal titration calorimetry (ITC) experiments in 20 mM phosphate buffer at pH 7.2 with 150 mM NaCl at 25 °C.

Isothermal titration calorimetry (ITC) provided the thermodynamic parameters (*K*, Δ*G*^0^, Δ*H*, and TΔ*S*^0^) that allow us to characterize how multivalency affects the binding affinities. ITC experiments were developed at pH 7.2 in 20 mM phosphate buffer with 150 mM NaCl at 25 °C. The stepwise addition of a solution of Fc-Lac **18** and **12**–**14** into a solution of Gal-3 led to a series of exothermic peaks with a decreasing area. Figure 3 shows a representative thermogram for the binding of **13** to Gal-3. Calorimetric data were fitted to an equal and independent binding site model facilitating the direct determination of the data displayed in Table 2. Globally, the complexation of the Fc-Lac derivatives with Gal-3 is enthalpy driven and entropically unfavorable, as the binding is dominated by enthalpy contributions (Figure 4).

**Figure 5:**
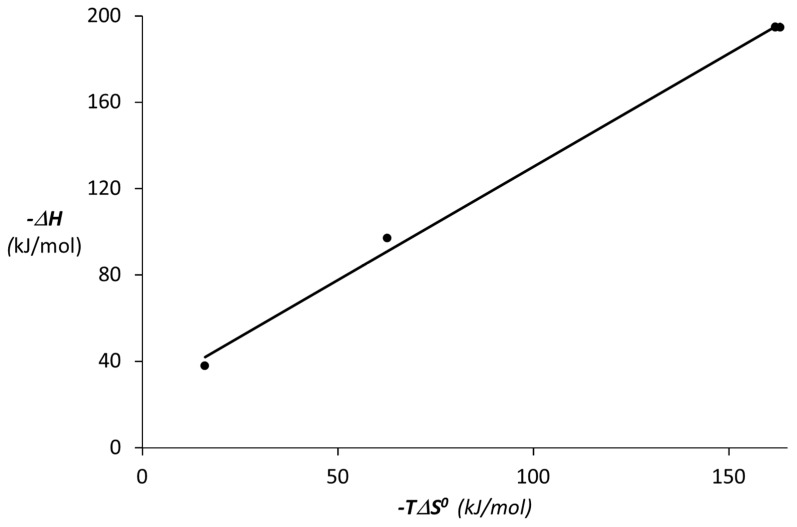
Enthalpy–entropy compensation plot. Plot of the −Δ*H* versus −*T*ΔS^0^ for the binding of compounds **12**–**14** and **18** to Gal-3. The plot shows a linear relationship with a slope of 1.05 and a correlation coefficient of 0.997.

As can be seen in Table 2, the *K* values increase with the number of lactoside residues of the dendrimers, from monomer **18** to hexadecamer **14**, due to a multivalent effect, with the binding affinities of tetramer **12**, octamer **13**, and hexadecamer **14** being −48-, −83-, and −156-fold higher than that for monomer **18**. A deeper analysis revealed that binding enthalpy Δ*H* values rapidly decrease (become more favorable) when going from monomer **18** to dendrimer **12**, which possesses four lactoside residues, where the Δ*H* value of **12** is −5 times lower than that for **18**. However, moving from tetramer **12** to octamer **13**, a small ΔΔ*H* value of −0.17 kJ mol^−1^ is observed, which likely means that not all the lactoside moieties are involved in the binding. Furthermore, the increase in the multivalence to 16 lactoside residues led to an increase of 97.81 kJ mol^−1^ (less favorable) of the enthalpy binding**.** Nevertheless, these changes in enthalpy binding with an increase in the number of lactoside residues are counterbalanced by changes in unfavorable entropic contributions. For example, a less favorable enthalpy binding for 16-mer **14**, compared to 4-mer **12** and 8-mer **13**, is compensated with a less unfavorable entropic term than that for the compounds. Thus, a strong enthalpy–entropy compensation behavior is observed with a slope slightly greater than unity (Figure 5), which is typically found in carbohydrate–lectin binding interactions [57,58]. Although the origin of this phenomenon remains unclear, such compensatory behavior has been attributed to solvent reorganization upon ligand binding, suggesting that the Fc-Lac conjugates follow a similar binding mechanism. The *n* values are lower than unity for all multivalent glycoconjugates (Table 2), as expected for the interactions of multivalent carbohydrates with oligomeric lectins [59]. Such behavior suggests an increase in the cross-linking between the glycoconjugates and Gal-3, with an increase of the number in lactoside residues. However, while Fc-Lac dendrimers **12** and **13** showed *n* values below and relatively close to the theoretically expected values for a tetramer (1/2 = 0.25) and an octamer (1/8 = 0.125), Fc-Lac 16-mer **14** showed a value larger than that theoretically expected (1/16 = 0.063), which indicates that, for this compound, not all of the lactoside residues participate in the binding, as noted above.

It is noteworthy to mention that the binding affinity of Fc-Lac dendrimers, and particularly hexadecamer **14**, is within the range of magnitude of the potent Gal-3 inhibitors [4,60]. Therefore, they can be applied as anti-Gal-3 agent for therapeutic purposes. We tested the binding ability of AuNP@Fc-Lac toward Gal-3 by following their UV–vis spectra variations after the addition of the lectin. It is well-known that this technique is a very useful tool to demonstrate protein-induced aggregation of AuNPs due to the dependence of the surface plasmon resonance (SPR) band on interparticle distance [36,61,62]. Thus, upon AuNPs aggregation, coupling interactions between the surface plasmon fields of the nanoparticles take place, leading to a change of the local refractive index around the nanoparticles, a red shift of the SPR band, and (usually) a broadening that, on occasion, results in absorbance decay.

**Figure 6:**
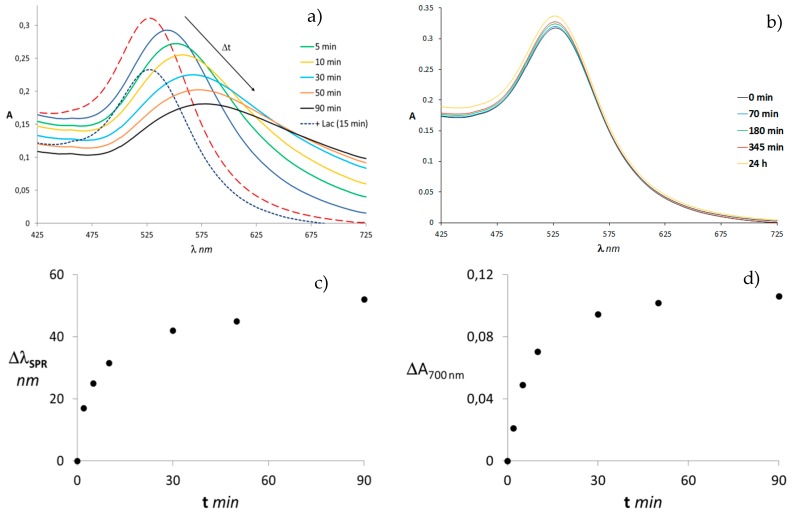
(**a**) UV–visible spectra of AuNP@Fc-Lac (2 nM) in a phosphate buffer of 10 mM, pH 7.2 with 20 mM NaCl in the presence of Gal-3 (10 µM) recorded at different times after addition of the lectin along with the UV–visible spectrum of this mixture after the addition of an aqueous saturated D-Lactose solution (dashed blue). (**b**) UV–visible spectra of AuNP@Fc-Lac (2 nM) in phosphate buffer 10 mM, pH 7.2, in the presence of BSA recorded at different times. Variation of Δλ_SPR_ (**c**) and absorbance at 700 nm (**d**) over time.

The treatment of the AuNP@Fc-Lac solution with Gal-3 in a phosphate buffer at pH 7.2 led to a red shift of the SPR band along with a progressive decrease of its absorbance (*A*_SPR_) with time (Figure 6). In this experiment, the used solution contained 20 mM NaCl in order to keep a high ionic strength and to ensure the stability of the protein and avoid its aggregation. Both the SPR band increase (Δλ_SPR_) and absorbance decrease (Δ*A*_SPR_) versus when time plots reached a plateau at 90 min. These results can be reasoned in terms of the closer special arrangement of AuNPs as a result of the formation of cross-linked complexes between AuNP@Fc-Lac and Gal-3. Thus, an increase in size of the aggregates with time brings about both a shift and a broadening of the SPR band. Eventually, the complex became water-insoluble and precipitated, leading to a decrease in absorbance. This UV–vis behavior of the binding interaction of Gal-3 and AuNP@Fc-Lac contrasts with that observed for the AuNP@Lac without a ferrocene moiety, which exhibited, in the presence of five times more concentrated Gal-3, a negligible change of λ_SPR_ and an increase of *A*_SPR_ during a period of 5 h [45]. Either the presence of the ferrocene moiety or the lower Gal-3 concentration at which the experiments were carried out (or both) seem to favor faster aggregation upon binding interactions, and allow for an arrangement of the AuNPs at more optimal interparticle distances. To test the participation of the lactose moiety in the binding, a saturated aqueous solution of lactose was added to the sample containing AuNP@Fc-Lac and Gal-3 after a period of incubation of 1.5 h. After 15 min, a completed re-dissolution was observed and the UV–vis spectrum showed the recovery of the original SPR band, albeit at a lower absorbance than the original. Moreover, the UV–vis spectrum of AuNP@Fc-Lac in the presence of bovine serum albumin (BSA) under the same conditions did not show any SPR band shift after a period of incubation of 5 h (Figure 6). These results confirm the specificity of the binding interaction and the lack of significance of the non-specific interactions.

### 3.4. Sensing Abilities Toward Gal-3

**Figure 7:**
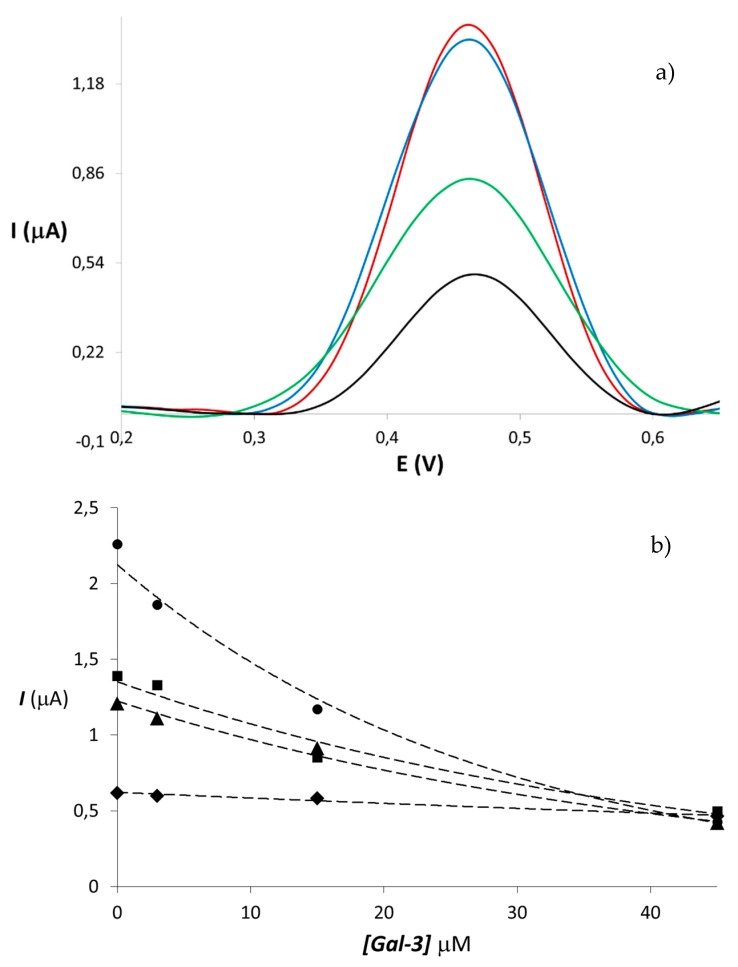
(**a**) Differential pulse voltammograms for compound **13** (12.5 µM) in the presence of increasing amounts of Gal-3 (0 µM, red; 3 µM, blue; 15 µM, 45 µM,) in 10 mM phosphate buffer at pH 7.2 with 20 NaCl. A decrease in the peak current was observed as the Gal-3 concentration increased. (**b**) Variation of the peak current intensity (DPV) of compounds **18** (50 µM, rhombs), **12** (25 µM, triangles), **13** (12.5 µM, squares), and **14** (6.25 µM, circles) in the presence of increasing amounts of Gal-3 (dashed lines represent the exponential fits).

**Figure 8:**
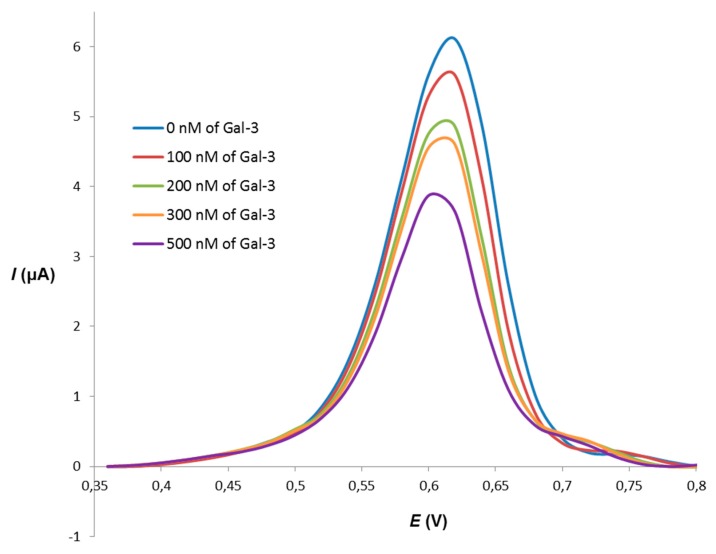
Differential pulse voltammogram for AuNP@Fc-Lac-modified glassy carbon electrode in the presence of increasing amounts of Gal-3 (0 nM, blue; 100 nM, red; 200 nM, green; 300 nM, orange; 500 nM, purple) in 10 mM phosphate buffer at pH 7.2 with 20 mM NaCl as the supporting electrolyte.

Once we had demonstrated the redox properties of PAMAM@Fc-Lac **12**–**14** and AuNP@Fc-Lac and their strong specific binding interactions with Gal-3, we studied their electrochemical sensing properties. In order to assess such sensing abilities, we evaluated the extent of the variation of the peak current upon the interaction of the electroactive spices with Gal-3. For this assessment, we used sensitivity parameters (*P*_s_), which are defined as (*I*_p0_−*I*_p_)/*I*_p0_, where *I*_p0_ and *I*_p_ represent the peak currents in the absence and in the presence of Gal-3, respectively. The *P*_s_ parameter is proportionally related to the number of redox units that became incorporated into the low-diffusing cross-linked complex upon binding with the lectin. Consequently, this parameter is related to both the stability of the complexes (*K* values) and the number of ferrocene units (valency) participating in the complex. In all cases, DPV voltammograms showed a progressive decrease of the *I*_p_ values as the Gal-3 concentration increased, which is indicative of the formation of electroactive Fc-Lac conjugate–Gal-3 complexes (Figure 7 and Figure 8). As the *I*_p_ value is proportional to the square root of the diffusion coefficient of the electroactive species, and the Fc-Lac-conjugate–Gal-3 complexes are expected to diffuse slower than the free Fc-Lac conjugate, the complexes have smaller *I*_p_ values than the uncomplexed species.

**Figure 9:**
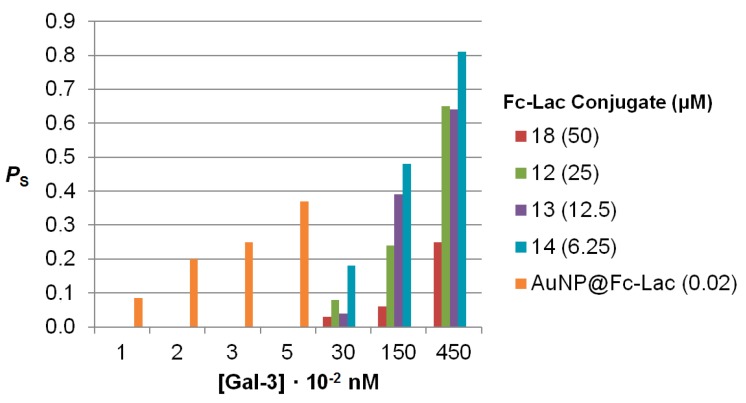
Sensitivity parameters (***P*_s_**) for ferrocene–lactose conjugates **12**–**14**, **18**, and AuNP@Fc-Lac with increasing concentrations of Gal-3.

**Figure 10:**
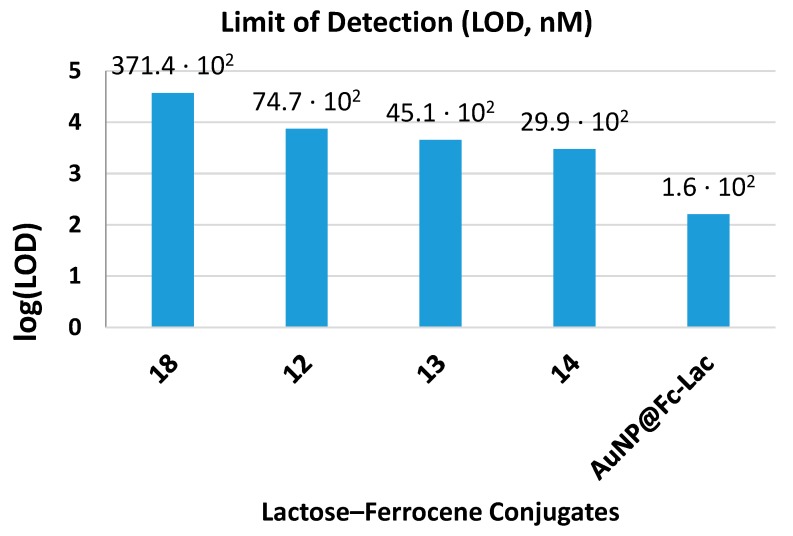
Limit of detection (LOD) for ferrocene–lactose conjugates **12**–**14**, **18**, and AuNP@Fc-Lac (logarithmic scale). The LOD (nM) value for each conjugate is shown over the respective bar.

The *P*_S_ of the Fc-Lac conjugates is shown in Figure 9. The highest sensitivity to Gal-3 is shown by AuNP@Fc-Lac, which is able to detect the lectin in nanomolar concentrations. The estimated limit of detection (LOD) for AuNP@Fc-Lac was calculated to be −160 nM, which is one and two orders of magnitude lower than that for PAMAM@Fc-Lac derivatives **12**–**14** and monomer Fc-Lac **18**, respectively (Figure 10). A 20 nM concentration of AuNP@Fc-Lac is slightly more sensitive than a 6.25 μM concentration of 16-mer **14** to detect Gal-3 at a concentration 15 times lower. Nevertheless, it should be noted that unlike the DPV measurements for **18** and **12**–**14**, those for AuNP@Fc-Lac involved electrode adsorption. Although the *P*_s_ values depend on the concentration of Gal-3, it can be observed that 16-mer **14** is the most sensitive PAMAM@Fc-Lac derivative in all tested Gal-3 concentrations. At a 45 μM concentration of Gal-3, which is above the LOD of the non-metallic Fc-Lac probes, glycodendrimer **14**, with 16 lactose units, is at a 6.25 μM concentration, 1.2-fold more sensitive than 4-mer **12** and 8-mer **13** at 25 and 12.5 μM concentrations, respectively. Likewise, the sensing ability of 16-mer **14** toward Gal-3, at the mentioned concentrations, is 3.2 times higher than that of the monovalent derivative in a solution eight times more concentrated. At lower 15 μM concentrations of Gal-3, the ratios of the *P*_s_ values for 16-mer **14** and 8-mer **13** are similar to those for a higher concentration of Gal-3, but the sensitivity of 4-mer **12** is −1.6-fold and twofold lower than that for 8-mer **13** and 16-mer **14**, respectively.

## 4. Conclusions

We have developed simple and convenient methods for the preparation of electrochemical probes for the high sensitivity detection of galectin-3. These probes consist of multivalent presentations of lactose–ferrocene conjugates based on poly (amido amine) dendrimers and gold nanoparticles, enabling enhanced galectin-3 binding affinity and multielectron exchange, resulting in an important improvement of electrochemical sensing abilities. Both lactosylferrocenylated dendrimers and gold nanoparticles showed a high affinity toward galectin-3 by calorimetric and optical methods, respectively, demonstrating that they bind to the lectin through a multivalent interaction, despite the monomeric nature of the lectin. Interestingly, the affinity of the multivalent conjugates, particularly those with higher valency, is within the range of magnitude of the most potent galectin-3 inhibitors, therefore, they can be applied as therapeutic agents. The highest sensitivity to galectin-3 by electrochemical methods was shown by lactosylferrocenylated gold nanoparticles, which were able to detect the lectin in nanomolar concentrations at least one order of magnitude lower than that needed by the dendrimers.

## Figures and Tables

**Table 1:** Oxidation potential (*E*_p_) and current intensity (*I*_p_) of differential pulse voltammetry (DPV) peaks for **18**, and **12**–**14** in 10 mM phosphate buffer at pH 7.2 and 25 °C with 20 mM NaCl.

Compound	Concentration (µM)	Ferrocene Concentration (µM)	*E*_p_ (V)	*I*_p_ (µA)
**18**	50	50	+0.292	0.62
**12**	25	100	+0.456	1.21
**13**	12.5	100	+0.461	1.39
**14**	6.25	100	+0.482	2.26

**Table 2:** Thermodynamic parameters for compounds **12**–**14** and **18** obtained by isothermal titration calorimetry in 20 mM phosphate buffer at pH 7.2 with 150 mM NaCl at 25 °C.

Compound	−ΔG^0^ (kJ∙mol^−1^)	−ΔH (kJ∙mol^−1^)	TΔS^0^ (kJ∙mol^−1^)	K·10^−4^ (M^−1^)	n
**18**	21.74 ± 0.11	37.81 ± 3.72	−16.07 ± 3.70	0.66 ± 0.03	1.00
**12**	31.48 ± 0.14	194.66 ± 7.30	−163.18 ± 7.25	31.82 ± 2.10	0.19 ± 0.05
**13**	32.90 ± 0.06	194.83 ± 2.01	−161.93 ± 1.98	54.82 ± 1.40	0.17 ± 0.01
**14**	34.36 ± 0.31	97.02 ± 3.60	−62.66 ± 3.20	103.04 ± 13.0	0.26 ± 0.01

## Supplementary Materials

The following are available online at https://www.mdpi.com/2079-4991/10/2/203/s1, UV–visible spectra for AuNPs@Fc-Lac preparation; transmission electron microscopy analysis; isothermal titration calorimetry experiments for compounds **18**, **12**, and **14**; differential pulse voltammetry titrations for compounds **18**, **12**, and **14** with galectin-3; sulfuric acid–phenol assays for compounds **12**–**14**; sensitivity parameters for **12**–**14**, **18**, and AuNP@Fc-Lac; ICP-OES measurements; mass spectra for compounds **12**–**14** and **17**; ^13^C-NMR and ^1^H-NMR spectra for compounds **12**–**14** and **17**.
