# Supplementary material for: Multivalent Lactose–Ferrocene Conjugates Based on Poly (Amido Amine) Dendrimers and Gold Nanoparticles as Electrochemical Probes for Sensing Galectin-3
Source: Nanomaterials (Basel). 2020 Jan 24;10(2):203. doi: 10.3390/nano10020203 (PMC7074905; doi:10.3390/nano10020203)
Supplement: Supplementary file 1 [file nanomaterials-10-00203-s001.pdf]

nanomaterials

Multivalent lactose–ferrocene conjugates based on poly(amido amine) dendrimers and gold nanoparticles as electrochemical probes for sensing Galectin-3

Manuel C. Martos-Maldonado, Indalecio Quesada-Soriano, Luis García Fuentes and Antonio Vargas-Berenguel*

Department of Chemistry and Physics, University of Almería, Carretera de Sacramento s/n, 04120 Almería, Spain; mmm588@inlumine.ual.es (M.C.M.-M.); indaqs@ual.es (I.Q.-S.); lgarcia@ual.es (L.G.-F.)
* Correspondence: avargas@ual.es

1. UV-visible spectra for AuNPs@Fc-Lac preparation

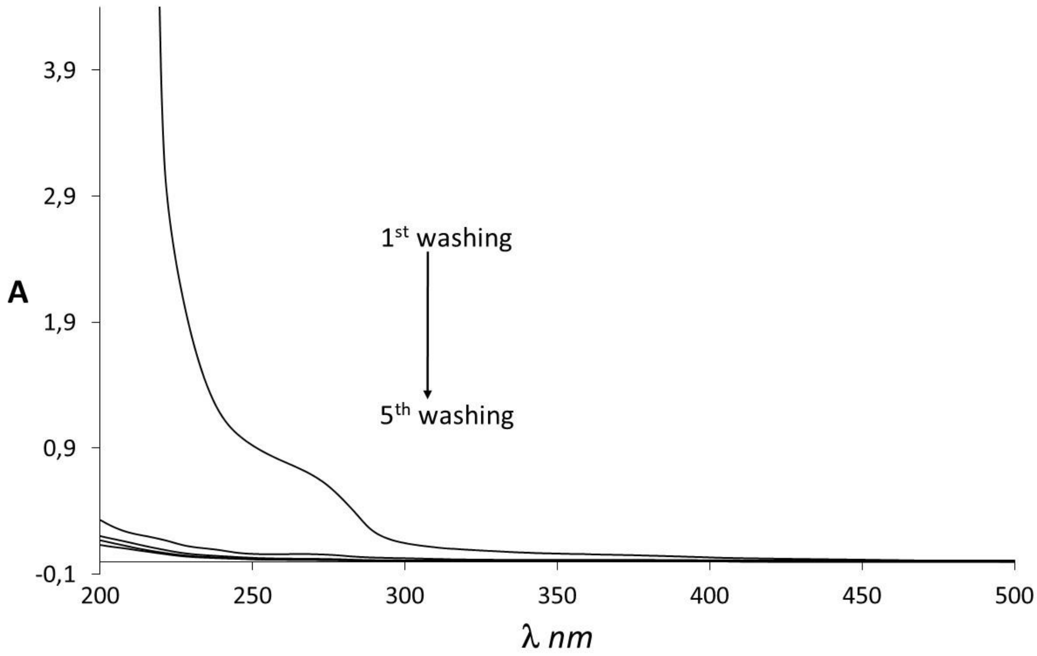

Figure S1. UV-visible spectra of ligand exchange washing solutions of **AuNPs@Fc-Lac** synthesis.

nanomaterials MDPI

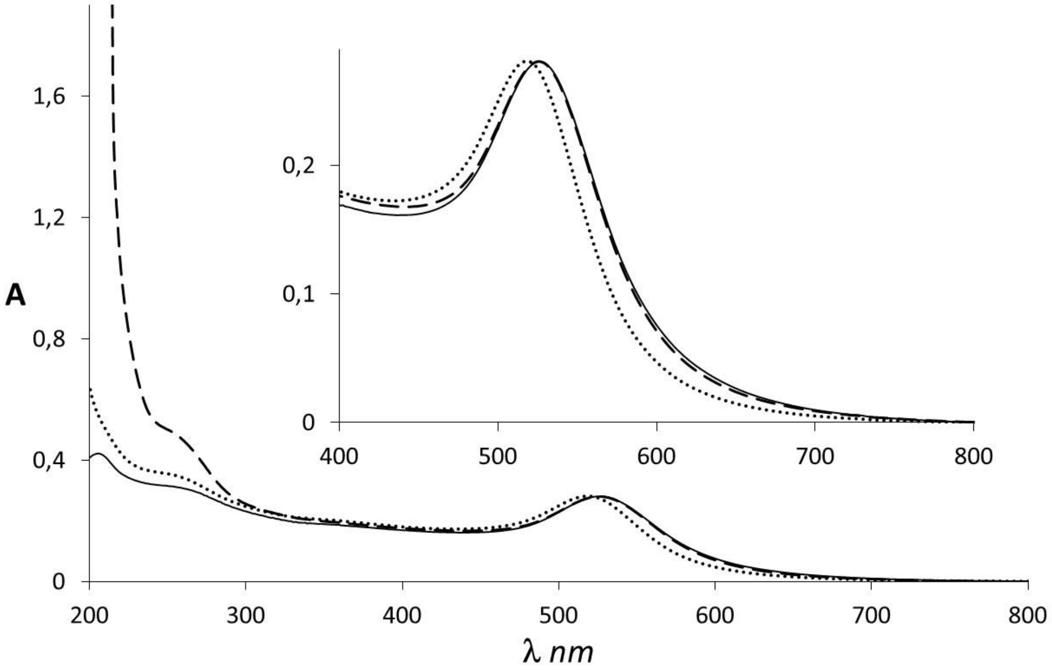

Figure S2. UV-visible spectra of citrate-stabilized AuNPs in 1 mM NaOH (···),**AuNPs@Fc-Lac** before removing the excess of ligand (— —) and **AuNPs@Fc-Lac** in a 10 mM phosphate buffer at pH 7.2 (—). Absorbances are normalized.

2. **Transmission electron microscopy analysis**

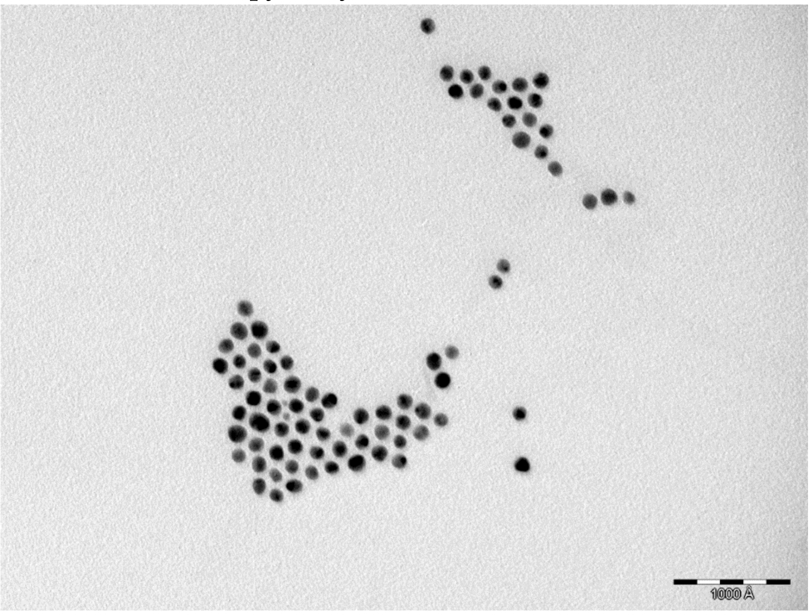

Figure S3. Representative TEM image of **AuNPs@Fc-Lac**.

Table S1. Diameters from measurements of individual particles.

	d (nm)		d (nm		d (nm		d (nm		d (nm
**1**	12.44	**21**	13.09	**41**	12.29	**61**	11.94	**81**	11.51
**2**	14.04	**22**	12.78	**42**	15.36	**62**	11.05	**82**	11.09
**3**	12.04	**23**	13.18	**43**	12.28	**63**	9.47	**83**	13.32
**4**	12.79	**24**	12.24	**44**	9.03	**64**	11.78	**84**	9.93
**5**	12.79	**25**	12.32	**45**	10.28	**65**	11.86	**85**	9.93
**6**	12.32	**26**	13.89	**46**	11.33	**66**	13.55	**86**	9.70
**7**	12.84	**27**	14.22	**47**	14.65	**67**	15.64	**87**	11.51
**8**	14.79	**28**	11.31	**48**	13.80	**68**	13.44	**88**	11.75
**9**	10.71	**29**	12.22	**49**	14.29	**69**	10.76	**89**	9.25
**10**	11.04	**30**	12.22	**50**	11.78	**70**	14.23	**90**	11.50
**11**	11.78	**31**	11.43	**51**	13.12	**71**	12.79	**91**	11.01
**12**	11.04	**32**	9.78	**52**	12.78	**72**	14.13	**92**	15.28
**13**	10.42	**33**	11.53	**53**	11.57	**73**	14.07	**93**	12.44
**14**	10.78	**34**	12.94	**54**	11.20	**74**	16.89	**94**	13.44
**15**	13.27	**35**	11.30	**55**	10.84	**75**	9.94	**95**	10.76
**16**	12.78	**36**	11.62	**56**	11.51	**76**	12.47	**96**	14.23
**17**	12.28	**37**	11.37	**57**	11.56	**77**	15.18	**97**	12.79
**18**	13.05	**38**	12.84	**58**	9.64	**78**	12.96	**98**	14.13
**19**	10.53	**39**	11.32	**59**	10.60	**79**	14.06	**99**	14.07
**20**	10.28	**40**	12.79	**60**	9.38	**80**	12.64	**100**	16.89

Average diameter = 12.25 ± 1.63

3. Isothermal titration calorimetry experiments for compounds 18, 12 and 14

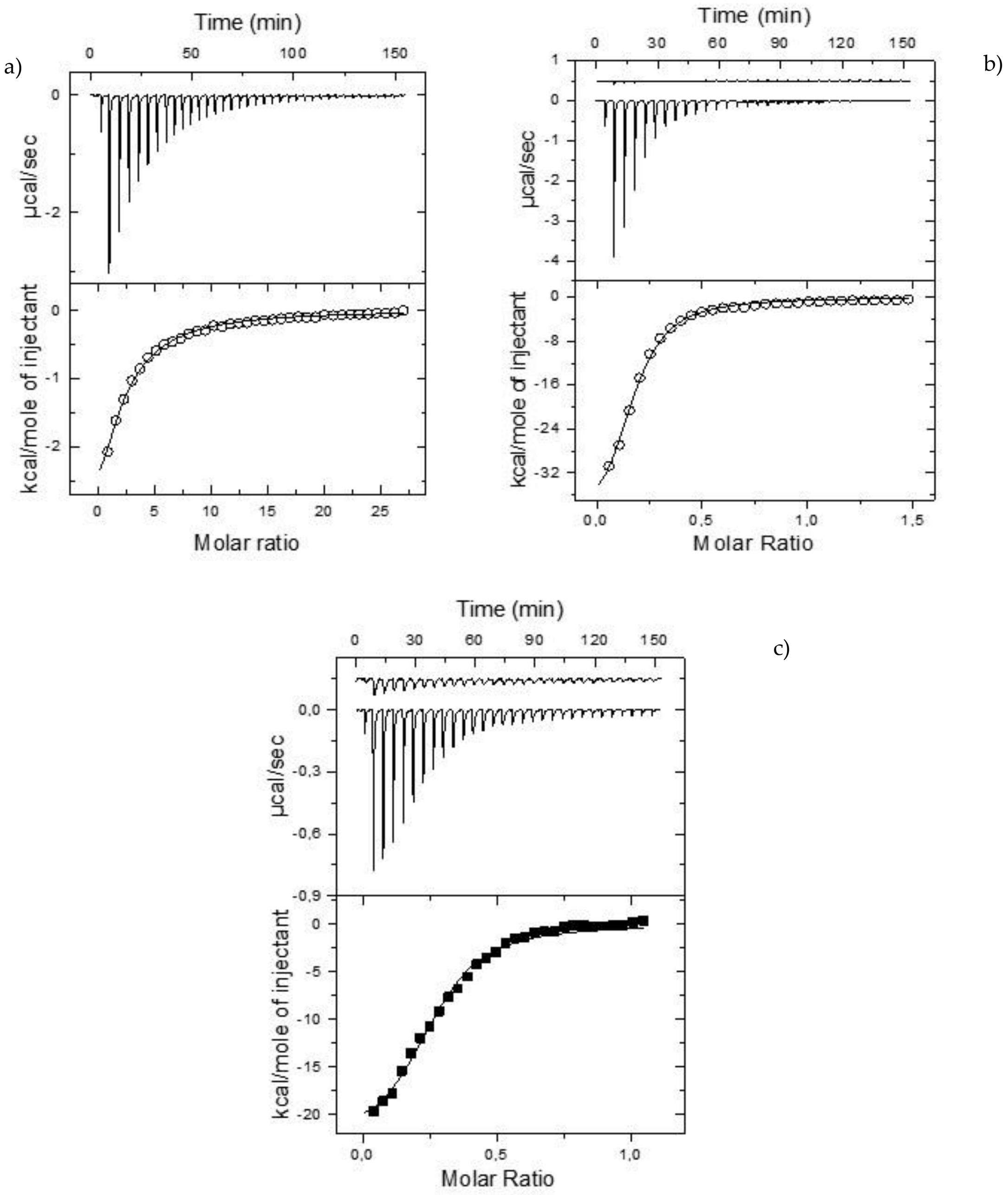

Figure S4. Titrations of Gal-3 with compounds **18** (a), **12** (b), **14** (c) in a 20 mM phosphate buffer at pH 7.2 with 150 mM NaCl at 25 °C. The top panel shows the raw calorimetric data, denoting the amount of generated heat (negative exothermic peaks) following each injection of the solution of conjugates (9.00 mM for **18**, 0.76 mM for **12**, and 0.21 mM for **14**) into the cell containing a solution of Gal-3 (45 µM for **18**, 55 µM for **12**, and 21 µM for **14**). The area under each peak represents the amount of heat released upon binding of the conjugates to the protein. The smooth solid lines represent the best fit of the experimental data to a model of *n* equal and independent sites.

4. Differential pulse voltammety titrations for compounds 18, 12 and 14 with Gal-3

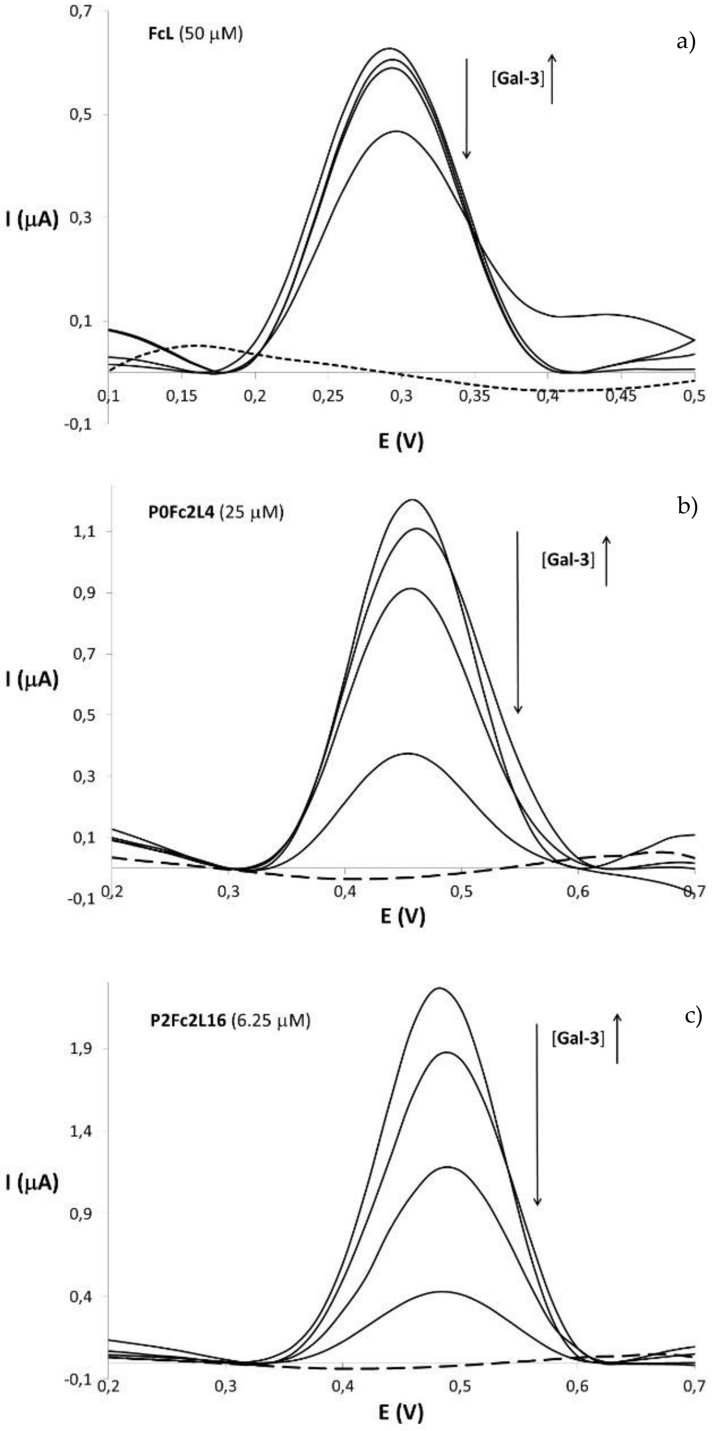

Figure S5. Differential pulse voltammograms for compounds **18** a) 50 µM, **12** b) 25 µM, and **14** c) 6.25 µM, in the presence of increasing amounts of Gal-3 (0-50 µM) in a 10 mM phosphate buffer at pH 7.2 with 20 mM NaCl.

nanomaterials

5. Sulfuric Acid-Phenol assays for compounds 12-14

A sulfuric acid-phenol assay[1] was used to determine the number of lactose units in compounds **12-14**. Briefly, concentrated sulfuric acid (600 μL) and a solution of 5 % phenol in water (120 μL) were added to an aqueous solution of lactose or compounds **12-14** (200 μL). The resulting solutions were incubated at 90 ºC for 5 min and then cooled down to room temperature in a water bath for another 5 min. Finally an UV-visible spectrum was recorded and the absorbance was measured at 487 nm. A calibration curve was done using lactose as reference (Figure S6). Lactose content in dendrimer solutions were obtained from calibration curve and the number of lactose units per dendrimer were estimated using data from table S2 and equation S1.

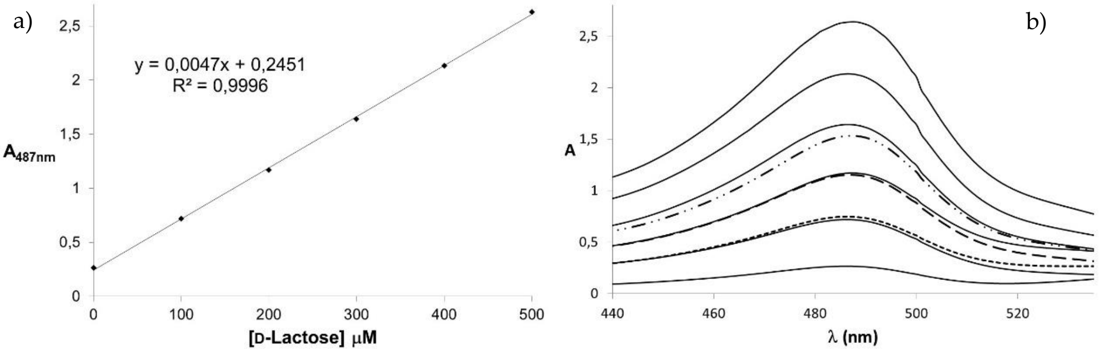

Figure S6.a) Calibration curve for lactose, and b) UV-visible spectra of lactose (—,100-500 μM), compounds **12** (— —), **13** (—··—) and **14** (---) solutions after being treated with sulfuric acid-phenol method.

Table S2. Sulphuric acid-phenol assays for dendrimers **12-14**.

	**12**	**13**	**14**
**A_{487nm}**	1.15252	1.53016	0.74766
**[Lac]**	193.07	273.42	106.93
**V(mL)**	50	50	50
**Mw_{core}**	837.04	2070.52	4537.53
**M_t (mg)**	8.5 ± 0.1	12.8 ± 0.1	5.1 ± 0.1
**M_{Lac} (mg)**	6.53	9.25	3.62
**n**	4.1 ± 0.2	8.0 ± 0.2	16.4 ± 1.1

Equation S1: $M_{Lac}/M_t = n \cdot Mw_{Lac}/(Mw_{core} + n \cdot Mw_{Lac}) \Rightarrow n = M_{Lac} \cdot Mw_{core}/(Mw_{Lac} \cdot (M_t - M_{Lac}))$

M_{Lac} = mass of lactose moieties; M_t = total mass; Mw_{core} = Molecular weight of alkynilated dendrimer core (black in Figure S7); Mw_{Lac} = Molecular weight of lactose moiety (red in Figure S7); n = number of lactose units per dendrimer.

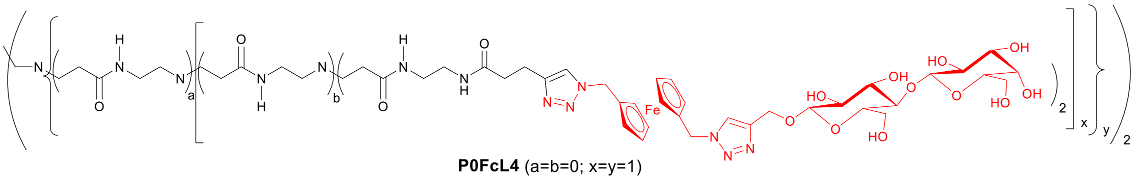

P0FcL4 (a=b=0; x=y=1)
P1FcL8 (a=0, b=1; x=2, y=1)
P2FcL16 (a=b=1; x=y=2)

Figure S7. Chemical structure of dendrimers **12-14**.

6. Sensitivity Parameters for 12-14, 18 and AuNP@Fc-Lac

Table S3. Sensitivity parameters (P_s) and limit of detection (LOD) for Ferrocene-Lactose Conjugates **12-14**, **18** and AuNP@Fc-Lac at increasing concentrations of Gal-3

Fc-Lac conjugates (μM)		P_s $[(I_{p0}-I_p)/I_{p0}]$							LOD ·10^{-2} nM
	**[Gal-3]**·10^{-2} (nM)	1	2	3	5	30	150	450	
**18** (50)						0.03	0.06	0.25	371.4
**12** (25)						0.08	0.24	0.65	74.7
**13** (12.5)						0.04	0.39	0.64	45.1
**14** (6.25)						0.18	0.48	0.81	29.9
AuNP@Fc-Lac (0.02)		0.085	0.20	0.25	0.37				1.6

The limit of detection (LOD) of **12-14, 18** and AuNP@Fc-Lac for Gal-3, defined as the lowest lectin concentration which gives a signal equivalent to three times the standard deviation of the peak current measured in the blank test solution, in the absence of lectin, was determined according to equation S4.

$$LOD = 3\sigma/b \qquad \text{eq. S4}$$

Where σ is the standard deviation of the electrochemical signals of the blank sample (from 3 measurements) and b is the slope of the linear calibration curve.[2]

7. ICP-OES measurements

The number of ligands per nanoparticles was determined as described elsewhere.[3] Aqua regia (1 mL) was added to a solution of **AuNPs@Fc-Lac** (≈0.7 nM) in MilliQ water (9 mL) and the resulting solution was sealed and stirred at 80 ºC for 20 min. The sample was cooled down and the content of gold and iron was determined by ICP- OES (Table S3). A blank sample was prepared treating in the same way MilliQ water.

Table S4. Concentration of gold and iron obtained for ICP-OES measurements for **AuNPs@Fc-Lac** solution.

	**[Au] ppb**	**[Au] μM**	**[Fe] ppb**	**[Fe] μM**
**Blank**	<LOD	<LOD	11.76 ± 0.10	
**AuNPs@Fc-Lac**	6654.63± 61.89	33.78 ± 0.31	70.59 ± 6.42	1.26 ± 0.12[a]

a) after substracting blank.

The concentration of AuNPs can be calculated from the concentration of gold according to equation S2, where 17 $Å^3$ is the volume of a gold atom[4]2,3 and r is the average radius of the AuNPs.

$$[AuNP] = [Au]\cdot 17\ Å^3/(4\cdot\pi\cdot r3/3) \text{ (equation S2)}$$

And the number of ligand per nanoparticle (N_{ligand}/AuNP) is given by equation S3 since each ligand has one iron atom.

$$N_{ligand}/AuNP = [Fe]/\ [AuNP] = [Fe]/([Au]\cdot 17\ Å^3/(4\cdot\pi\cdot r^3/3)) \text{ (equation S3)}$$

Introducing an r value of 61.25 Å as determined by TEM analysis (see above), and the data obtained from ICP-OES measurements we obtained 1765 ± 32 ligand per nanoparticle for **AuNPs@Fc-Lac**.

8. Mass spectra for compounds 12-14 and 17

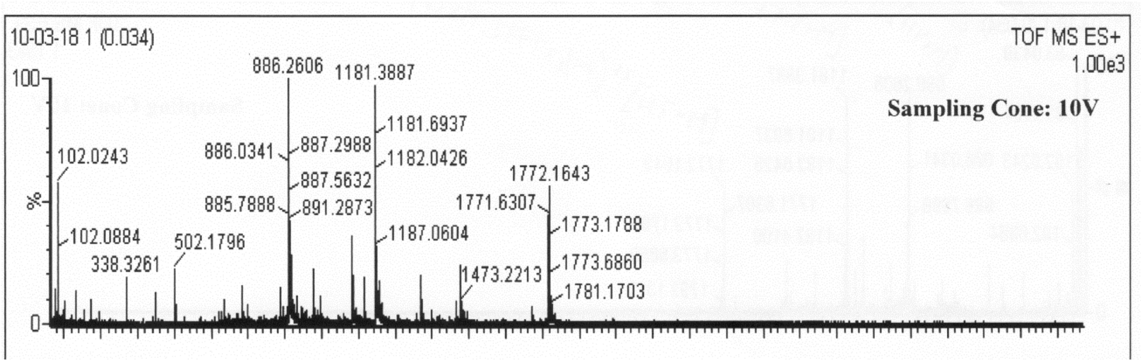

Figure S8. ESI-TOF spectrum for compound **12**.

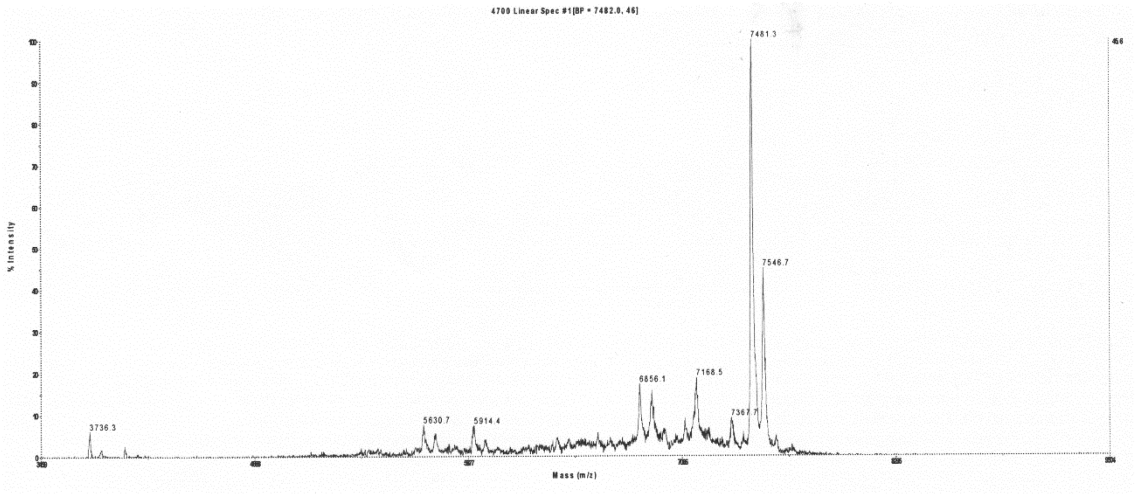

Figure S9. MALDI-TOF spectrum for compound **13**.

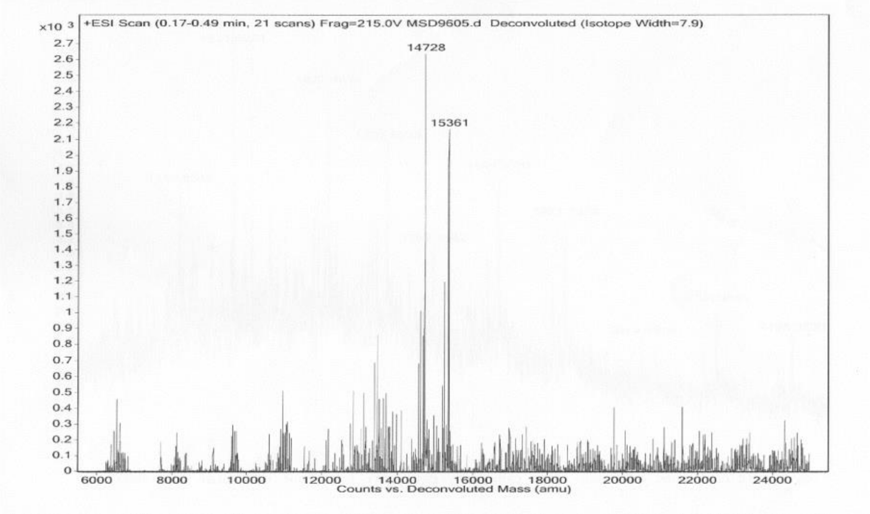

Figure S10. MALDI-TOF spectrum for compound **14**.

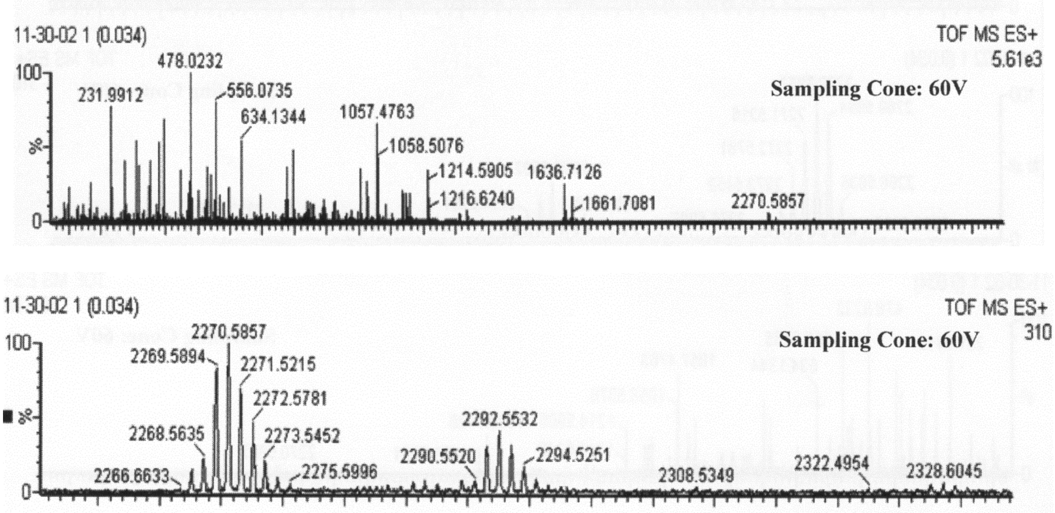

Figure S11. ESI-TOF spectrum for compound **17**.

9. ^{13}C-NMR and ^{1}H-NMR spectra for compounds 12-14 and 17

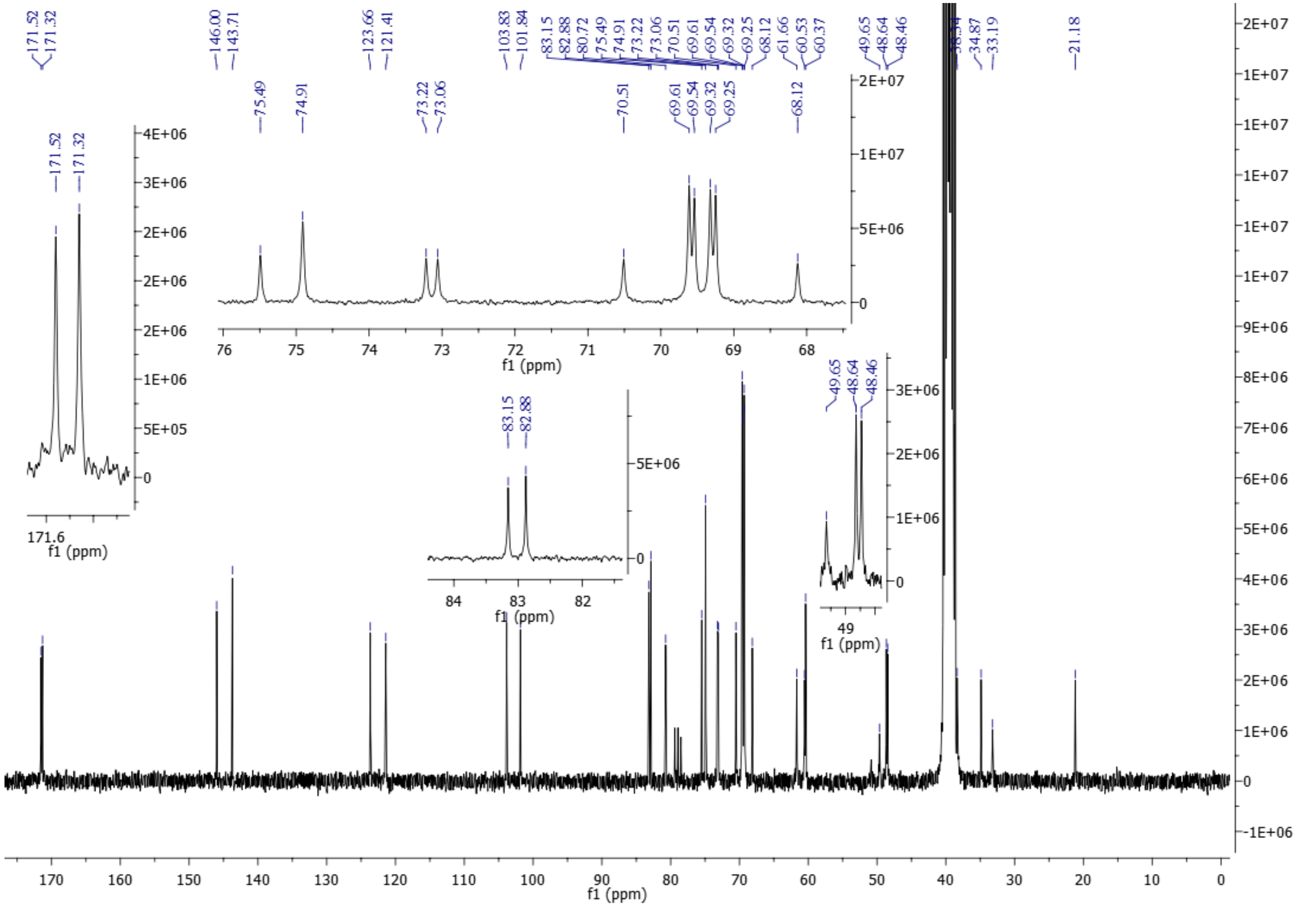

Figure S12. ^{13}C-NMR spectrum (75 MHZ, DMSO-d_6, 25 ºC) for compound **12**.

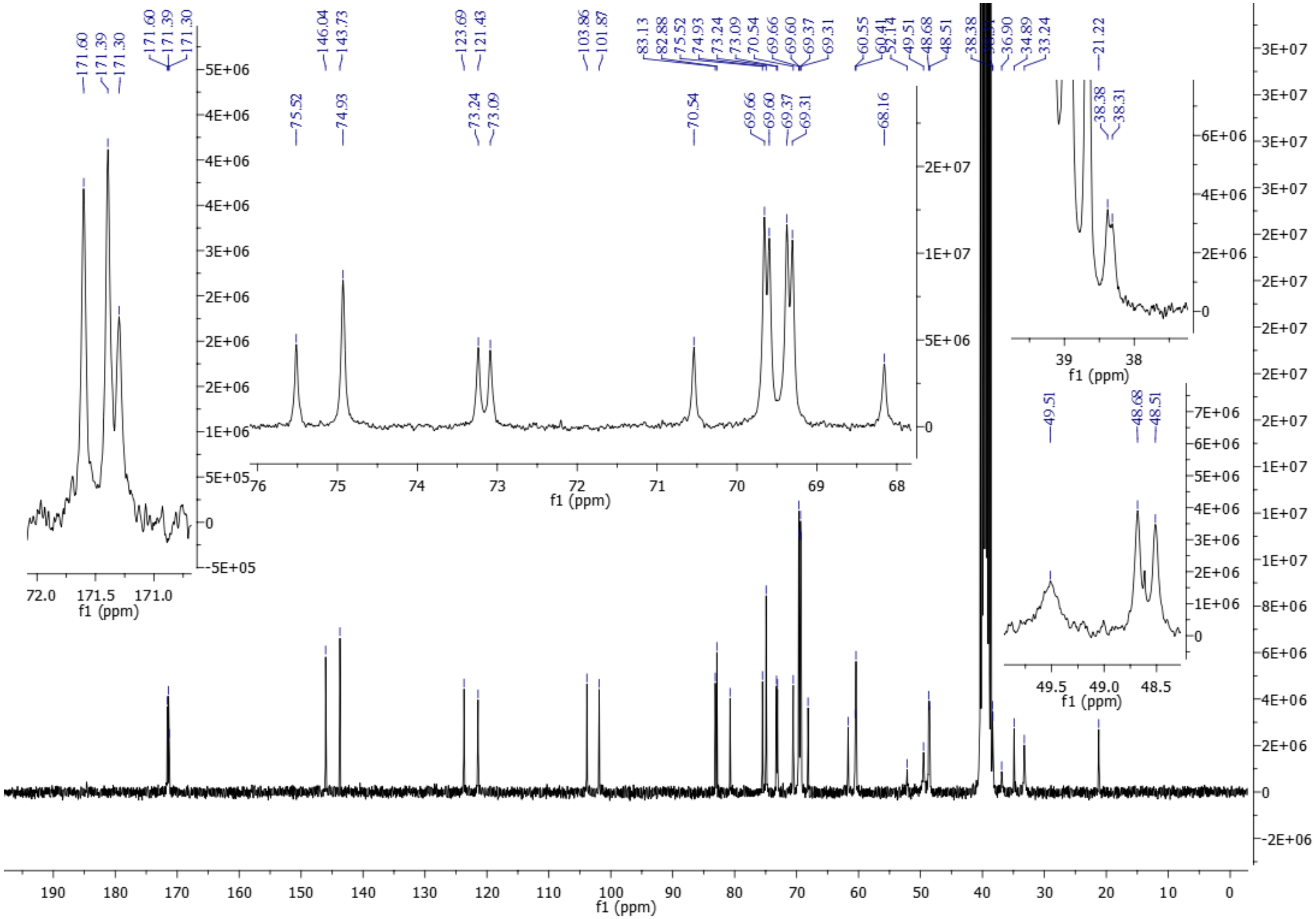

Figure S13. ^{13}C-NMR spectrum (75 MHZ, DMSO-d_6, 25 ºC) for compound **13**.

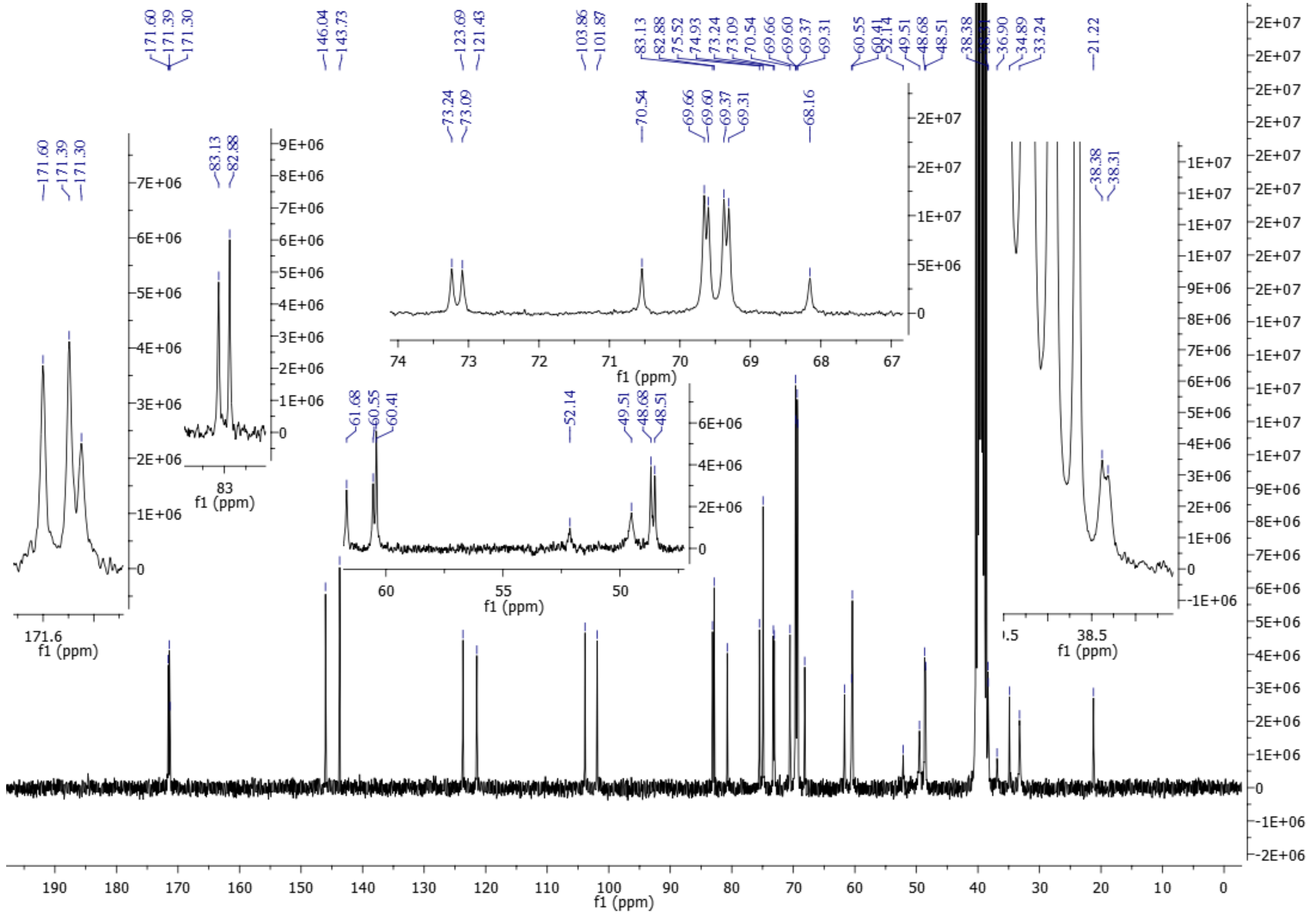

Figure S14. ^{13}C-NMR spectrum (75 MHZ, DMSO-*d*6, 25 ºC) for compound **14**.

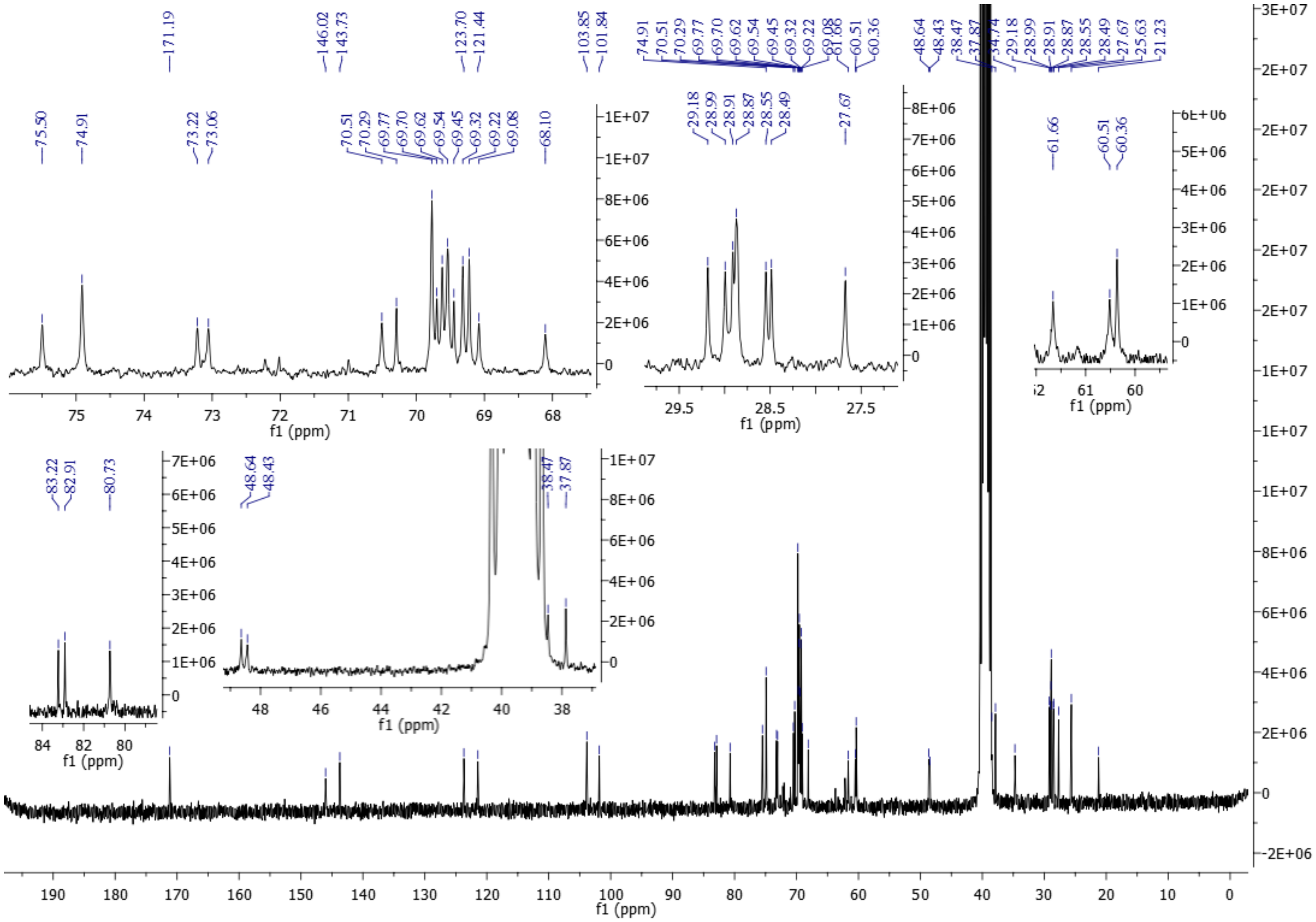

Figure S15. ^{13}C-NMR spectrum (75 MHZ, DMSO-*d*$_6$, 25 ºC) for compound **17**.

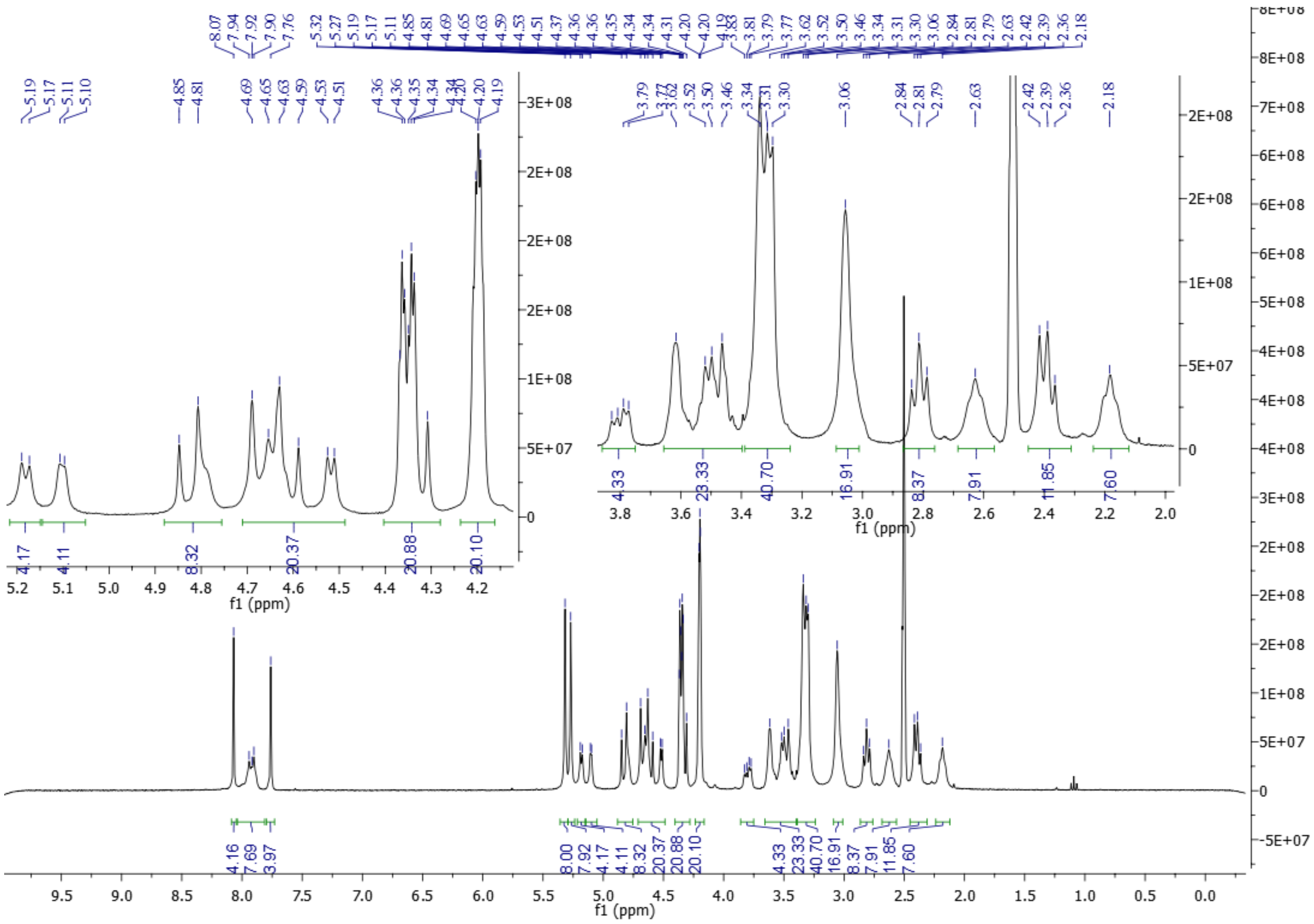

Figure S16. ^{1}H-NMR spectrum (300 MHZ, DMSO-d_6, 25 ºC) for compound **12**.

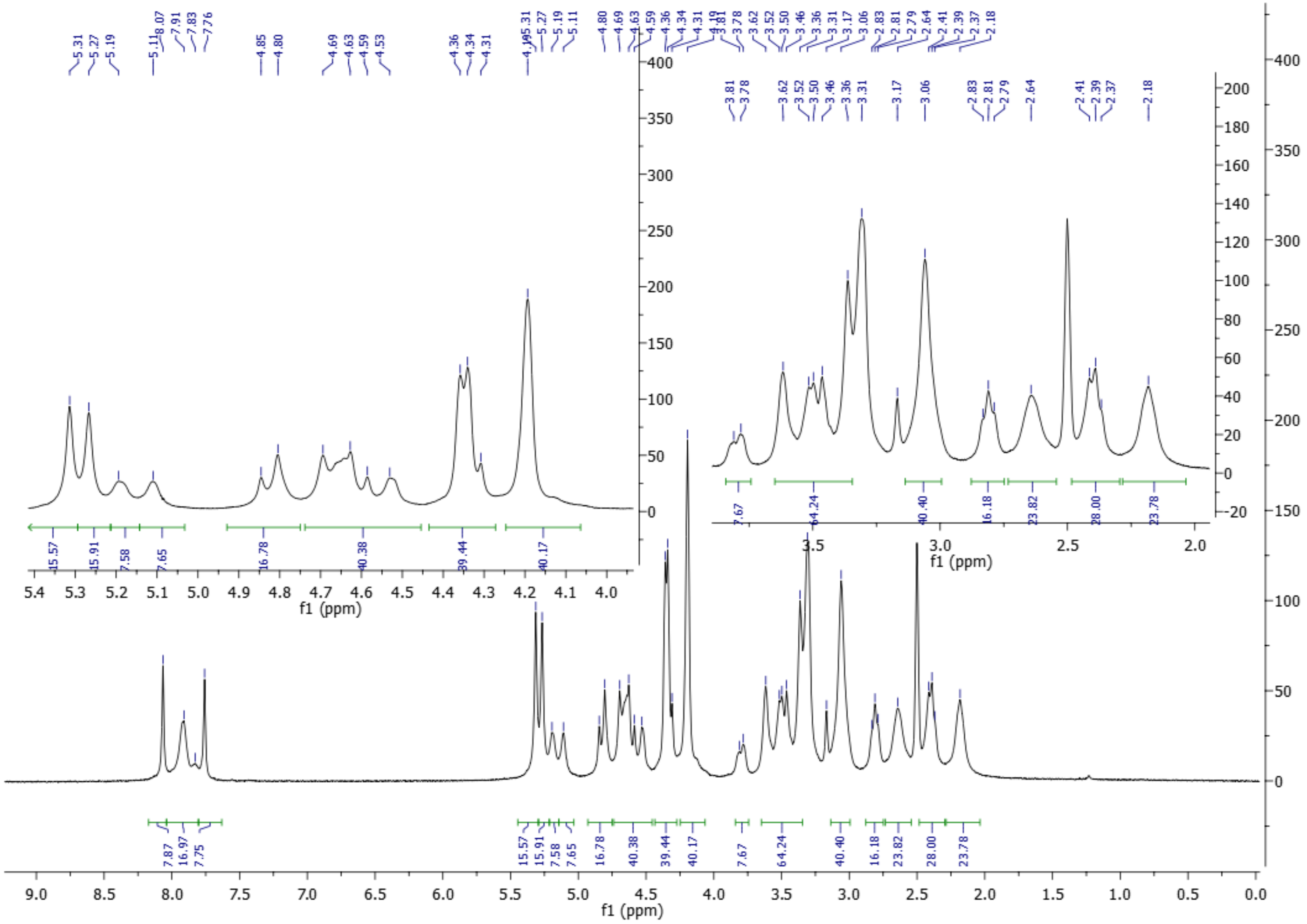

Figure S17. ^{1}H-NMR spectrum (300 MHZ, DMSO-d_6, 25 ºC) for compound **13**.

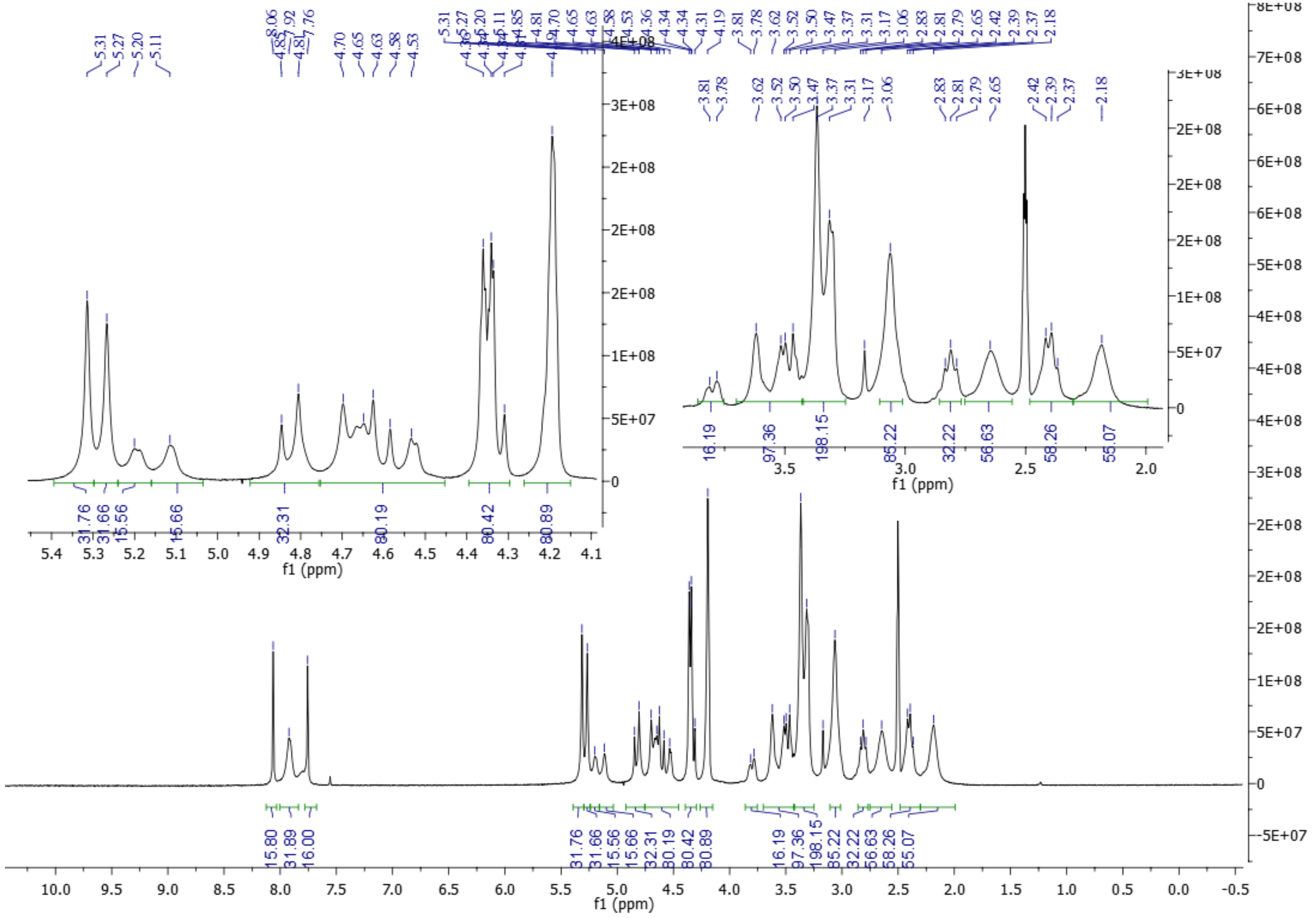

Figure S18. ^{1}H-NMR spectrum (300 MHZ, DMSO-d_6, 25 ºC) for compound **14**.

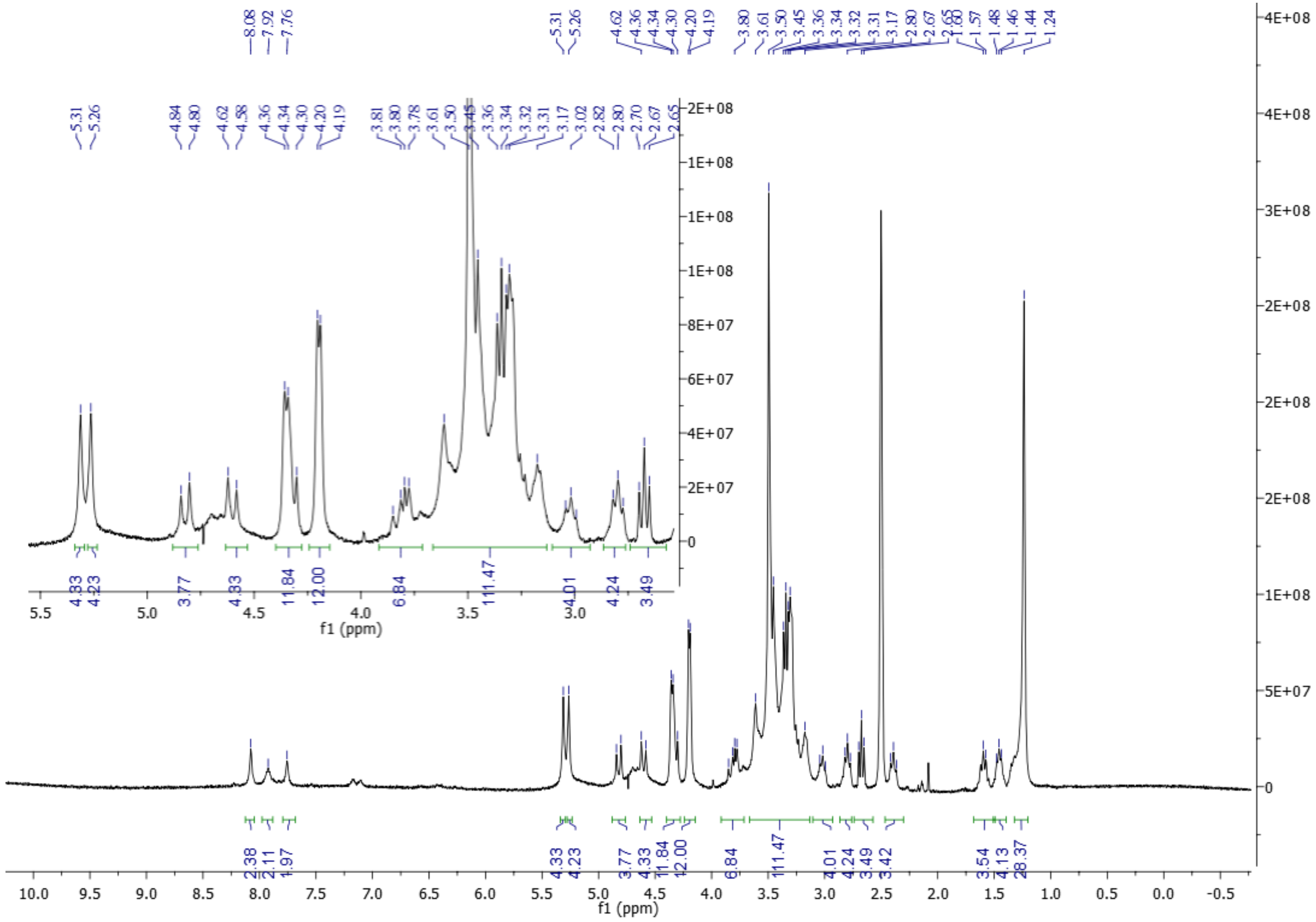

Figure S19. ^{1}H-NMR spectrum (300 MHZ, DMSO-d_6, 25 ºC) for compound **17**.

[1] M.C Martos-Maldonado, J.M. Casas-Solvas, I. Quesada-Soriano, L. García-Fuentes, A. Vargas-Berenguel. *Langmuir* **2013**, *29*, 1318.
[2] D. MacDougall, W. B. Crummett, *Anal. Chem.* **1980**, *52*, 2242.
[3] M.C. Martos-Maldonado, M.B., Thygesen, K. J. Jensen, A. Vargas-Berenguel, *Eur. J. Org. Chem.* **2013**, 2793.
[4] a) D.V. Leff, P.C. Ohara, J.R. Heath, W.M. Gelbart, *J. Phys. Chem.* **1995**, *99*, 7036. b) M.C. Daniel, J. Ruiz, S. Nlate, J.C. Blais, D. Astruc, *J. Am. Chem. Soc.* **2003**, *125*, 2617.
